# *TOB1* suppresses proliferation in *K‐Ras* wild‐type pancreatic cancer

**DOI:** 10.1002/cam4.2756

**Published:** 2019-12-31

**Authors:** Yuru Bai, Lu Qiao, Ning Xie, Yan Li, Yongzhan Nie, Yan Pan, Yupeng Shi, Jinhai Wang, Na Liu

**Affiliations:** ^1^ Department of Gastroenterology the Second Affiliated Hospital of Xi'an Jiaotong University Xi'an Shaanxi China; ^2^ Shaanxi Key Laboratory of Gastrointestinal Motility Disorders the Second Affiliated Hospital of Xi'an Jiaotong University Xi'an Shaanxi China; ^3^ Department of Geriatric Respiratory and Endocrinology (The Third Unit of Cadre's Ward) the Second Affiliated Hospital of Xi'an Jiaotong University Xi'an Shaanxi China; ^4^ State Key Laboratory of Cancer Biology the Fourth Military Medical University Xi'an Shaanxi China; ^5^ Xijing Hospital of Digestive Diseases Xijing Hospital the Fourth Military Medical University Xi'an Shaanxi China

**Keywords:** calcium pathway, pancreatic cancer, proliferation, *TOB1*

## Abstract

*TOB1* participates in various kinds of cancers. However, its role in pancreatic cancer has rarely been reported. In this study, we explored the expression and mechanisms of *TOB1* in regulating the malignant phenotype of pancreatic cancer cells. *TOB1* expression was determined by data mining and immunohistochemistry (IHC), and its localization was observed by immunofluorescence. CCK‐8 cell proliferation, colony formation, flow cytometric, transwell migration, and Western blot (WB) assays were used to examine how it impacts the malignant phenotype of pancreatic cancer. Furthermore, *Foxa2* binding to *TOB1* was tested by dual‐luciferase reporter assays, and RNA‐Seq was performed to identify signaling pathways. We found *TOB1* was downregulated in pancreatic cancer tissues and was mainly located in the cytoplasm. *TOB1* overexpression reduced the proliferation of *K‐Ras* wild‐type pancreatic cancer cells but made no difference to cell migration and invasion. *Foxa2* overexpression significantly enhanced *TOB1* promoter activity. Moreover, overexpressing *TOB1* substantially enriched the calcium pathway in *K‐Ras* wild‐type pancreatic cancer cells. In conclusion, *TOB1* may suppress the proliferation of *K‐Ras* wild‐type pancreatic cancer cells by regulating calcium pathway genes.

## Graphical abstract

*Foxa2*, as an upstream transcription factor, may regulate *TOB1* expression. *TOB1* overexpression decreased expression of Cyclin D1, *CDK2* and *CDK4*, and caused proliferation inhibition of *K-Ras* wild‐type pancreatic cancer cells, at least in part through the calcium pathway.

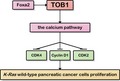

## 1 INTRODUCTION

Pancreatic cancer ranks fourth among cancer‐related deaths in the United States,^1^ and will be second in 2030.^2^ Over the past decades, pancreatic cancer has also been in the top 10 of all malignant tumors in China.^3^ Due to a lack of early symptoms, over half patients had a 5‐year survival of 3%.^1^ Pancreaticoduodenectomy is a potentially curative treatment, but less than 20% of patients have the opportunity to undergo surgery.^4^ Pancreatic cancer remains a challenging, noncurable disease. Therefore, there is a pressing need for identification of effective treatments based on deeper exploration of the potential mechanisms of this cancer to improve survival.

The regulation of cell proliferation is crucial to the activation of important cellular processes. Cell hyperproliferation is a significant biological feature of tumors.^5^ In the early 1990s, the Tob/BTG antiproliferative (APRO) protein family was found, which could negatively control tissue growth and development.^6^ *TOB1* was first found to interact with ErbB‐2 (also known as HER‐2) in a human breast cell line.^7^ Research has shown that *TOB1* has vital roles in embryogenesis, activation of T cells, bone formation, and metabolism.^8^ The roles of *TOB1* in cancer became apparent when mice lacking *TOB1* were observed to spontaneously develop tumors.^9^ Subsequent studies reported that reduced *TOB1* expression or inactivation by phosphorylation contributed to carcinogenesis and tumor growth.^10^, ^11^, ^12^ Studies have indicated that regulation of nuclear localization of *TOB1* also affects its antiproliferative activity,^13^, ^14^ but this conclusion is still unclear. Other reports revealed that *TOB1* participated in regulating the proliferation, invasion, metastasis, and resistance of some cancers.^15^, ^16^, ^17^ Furthermore, *TOB1* may predict the prognosis of node‐negative breast cancer.^18^

Accumulating evidence has shown that *TOB1* functions as a tumor suppressor. However, few research have examined the expression and biological role of *TOB1* in pancreatic cancer. In this study, we found that *TOB1* is mainly located in the cytoplasm. Low expression of *TOB1* was detected in pancreatic cancer tissues and inversely correlated with tumor size. *TOB1* overexpression prevented the cell transition from G_1_ phase to S phase, and caused the proliferation of *K‐Ras* wild‐type pancreatic cancer cells was inhibited, at least in part through the calcium pathway. Furthermore, a luciferase reporter assay indicated that *Foxa2*, as an upstream transcription factor, regulates *TOB1* expression.

## 2 MATERIALS AND METHODS

### 2.1 Clinical samples

We purchased two pancreatic cancer tissue microarrays from Shanghai Outdo Biotech: HPanA030PG02 contains 15 cases of pancreatic ductal adenocarcinoma (PDAC) and their adjacent noncancerous tissues (NCTs) with one point for each tissue; and HPan‐Ade180Sur‐02 contains 80 cases of matched cancer/paracancerous samples and 20 cases of unpaired cancer tissues with one point for each tissue. All patients in HPan‐Ade180Sur‐02 were followed up from 1 to 87 months after the operation, and all specimens involved were determined by HE staining.

### 2.2 Cell lines and culture

The human pancreatic cancer cell lines BxPC‐3 and Patu8988‐t were cultured with RPMI‐1640 medium (HyClone) containing 10% fetal bovine serum (FBS, Gibco) at 37°C in 5% CO_2_. All cell lines were authenticated by the China Center for Type Culture Collection (CCTCC) using short tandem repeat (STR) analysis.

### 2.3 Oncomine database analysis

Oncomine (http://www.oncomine.org) was utilized to analyze the mRNA expression difference of *TOB1* in pancreatic cancer and normal pancreatic tissues. The thresholds were set as follows: gene: *TOB1*; analysis type: cancer vs normal analysis; data type: mRNA; sample type: clinical specimen; *P* value: .05; fold change: 2; gene rank: all. The original expression data were collected to draw a box plot.

### 2.4 Immunohistochemistry

This method was reported in a previous study.^19^ Sections were incubated with monoclonal rabbit anti‐*TOB1* (14915‐1‐AP, Proteintech Technology) at 1:100 dilution. According to semiquantitative assessment of the staining intensity and the percentage of marked tumor cells, *TOB1* expression was evaluated as absent (−), weak (+), moderate (++), or strong (+++).

### 2.5 Lentivirus transfection

All lentiviruses we used were purchased from Shanghai Genechem. First, cells were transduced at a multiplicity of infection (MOI) value of 10, 20, 50, or 100 under different transfection conditions for the preliminary experiments. The results showed that cells in the transfected group with MOI values of 10 and 20 did not show green fluorescence, while those in the group with values of 50 and 100 had stronger fluorescence (Figure S1). We chose an MOI value of 50 for the formal experiment. BxPC‐3 and Patu8988‐t cells were seeded at 1.5 × 10^5^ cells/well and 0.5 × 10^5^ cells/well into 6‐well plates, respectively. Cells were transduced at an MOI value of 50. Viruses and cells were incubated in enhanced infection solution with a final volume of 1 mL. Twenty‐four hours later, viruses were removed, cells were rinsed twice with phosphate buffered saline (PBS), and fresh RPMI‐1640 medium supplemented with 10% FBS was added. Puromycin (2.5 µg/mL for BxPC‐3 and 5 µg/mL for Patu8988‐t) was added at 72 hours posttransfection, and cells were cultured under this condition for a month. Cells of the negative control group were established under the same conditions. The efficiency of transfection was verified using a fluorescence microscope and Western blot (WB) analysis. After verification, puromycin was reduced to half of the selection dose for long‐term culture.

### 2.6 Immunofluorescence

Cells were seeded at 5 × 10^4^ cells/well into a Millicell EZ SLIDE (Millipore). The next day, cells were washed twice with PBS, each time for 2 minutes, before being fixed with 4% paraformaldehyde (PFA) for 30 minutes, followed by washing with PBS for 15 minutes. Then, the cells were treated with 0.3% Triton X‐100 PBS for 30 minutes, washed with PBS for 15 minutes, and incubated with 5% BSA for 30 minutes. Anti‐*TOB1* antibody was diluted 1:50 at 4°C overnight. After the cells were washed with PBS three times for 5 minutes each, in the dark conditions, secondary antibodies conjugated with Alexa Fluor 594 goat anti‐rabbit IgG (Invitrogen) were loaded into wells at room temperature (RT) for 60 minutes. Cells were washed as before, followed by DAPI (Beyotime) for 3 minutes. Prior to the end of the immunostaining process, PBS was used to wash the cells again. Immunofluorescence images were harvested with a laser scanning confocal microscope (Lecia).

### 2.7 Cell proliferation assay

The Cell Counting Kit‐8 assay (Dojindo) was used to measure the proliferation of different cell lines according to the manufacturer's instructions. 3000 BxPC‐3 cells and 2000 Patu8988‐t cells were plated in 100‐μL medium in 96‐well plates in quintuplicate. Cells were incubated in normal culture medium containing 10% CCK‐8 for 2 hours at 37℃ in 5% CO_2_. The optical density values were determined by reading the absorbance at 450 nm.

### 2.8 Colony formation assay

Patu8988‐t and BxPC‐3 were seeded in 6‐well plates at a density of 500/well and 1000/well, respectively. About 7‐10 days later, after fixing with 4% PFA for 30 minutes, we stained cells with 0.05% crystal violet for 20 minutes at RT. Finally, the number of colonies was counted, with a colony of at least 50 cells counted as one colony.

### 2.9 Cell cycle analysis

Single‐cell suspensions were collected. After washing twice with cold PBS, we fixed cells with cold 75% ethanol and stored them at −20°C for over 24 hours. Cells were washed with cold PBS twice, then 1 × 10^6^ cells were resuspended in 1‐mL PI/RNase staining solution (BD Biosciences), and finally analyzed by the FACSCalibur flow cytometer (BD Biosciences).

### 2.10 Transwell migration assay

1 × 10^5^ cells of Patu8988‐t and BxPC‐3 cells were resuspended in medium without FBS and plated in the upper chambers of Transwell plates (8‐μm, Corning), while the lower chambers were loaded with 10% FBS medium (Patu8988‐t) or 20% FBS medium (BxPC‐3) as a chemoattractant. Cells were incubated for 24 hours (Patu8988‐t) or 48 hours (BxPC‐3). After fixing with 4% PFA for 30 minutes, cells were stained with 0.05% crystal violet for 20 minutes. Cells from five regions were counted under a microscope using 100× magnification.

### 2.11 Western blot analysis

The protocol was reported in a previous study.^19^ The antibodies we used were as follows: anti‐E‐cadherin (at 1:2000, #3195, Cell Signaling Technology [CST]), anti‐N‐cadherin (at 1:2000, #13116S, CST), anti‐beta‐catenin (at 1:5000, #8480, CST), anti‐Vimentin (at 1:2000, ab92547, Abcam), anti‐*CDK2* (at 1:250, #2546S, CST), anti‐*CDK4* (at 1:250, #12790S, CST), anti‐cyclin D1 (at 1:2000, 60186‐1‐Ig, Proteintech Technology), anti‐*TOB1* (at 1:1500), and the corresponding secondary antibodies (at a 1:50 000, MYBiotech). For verification of equal protein loading, polyclonal rabbit anti‐GAPDH (at 1:6000, 10494‐1‐AP, Proteintech Technology) was used as a loading control. ImageJ software was used to analyze and quantify each band and relative band intensities.

### 2.12 Luciferase reporter assay

Fragments of the *TOB1* (NM_001243877) promoter were cloned into the pGL3‐Basic Vector to generate promoter‐reporter constructs. All constructs were verified by sequencing. Cells were seeded in 24‐well plates and transiently transfected with plasmids containing firefly luciferase reporters and recombinant promoter‐reporter constructs. Reporter constructs containing the full‐length *TOB1* promoter region and other truncated fragments were transiently transfected into *Foxa2*‐overexpressing cells (transiently transfected with plasmids) and the respective control cells. The luciferase activity was measured after incubation for 48 hours using the Dual‐Luciferase Reporter Assay Kit (Promega). The transfection efficiency was normalized with Renilla luciferase activity. The promoter sequence of *TOB1* was listed in Supporting Information.

### 2.13 RNA extraction and RNA‐Sequencing (RNA‐Seq)

Total RNA was extracted from four cell lines, BxPC‐3‐LV‐*TOB1*, BxPC‐3‐LV‐NC, Patu8988t‐LV‐*TOB1*, and Patu8988t‐LV‐NC, using TRIzol^®^ Reagent (Invitrogen) according to the manufacturer's instructions. NanoPhotometer spectrophotometer and Agilent 2100 Bioanalyzer were applied to evaluate the quality of the RNA samples. The RNA‐Seq service was provided by Beijing Novogene. What tools used in this work contained Illumina HiSeq™ 4000 system (Illumina), the FastQC tool and the HISAT2 tool.

### 2.14 Statistical analyses

Each experiment should be repeated independently at least three times. SPSS statistical software (Version 21.0) was used to conduct statistical analyses. Images were obtained using GraphPad Prism software (Version 7.0). The counting data are represented by frequency or percentage, and the measurement data are expressed as the mean ± standard deviation (SD). The rank sum test was used to reveal correlation between *TOB1* expression and clinicopathologic features. The overall survival (OS) curve was plotted by Kaplan‐Meier and compared with the log‐rank test. The differences between the two groups were assessed using Student's *t* test. *P* < .05 was accepted as indicative of significant differences.

## 3 RESULTS

### 3.1 *TOB1* expression is downregulated in pancreatic cancer

**Figure 1:**
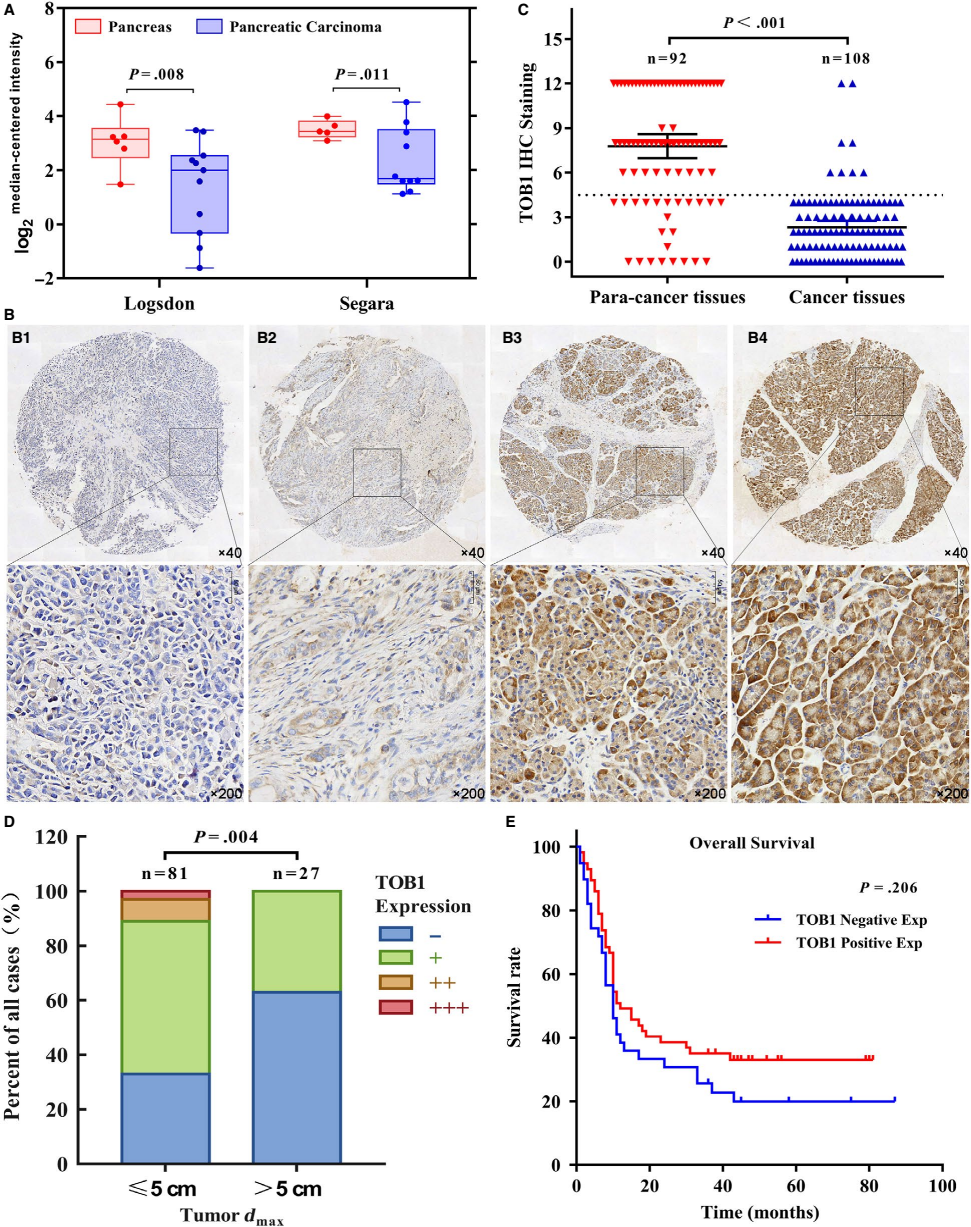
Expression and clinical significance of *TOB1* in pancreatic cancer. A, *TOB1* mRNA is reduced in pancreatic carcinoma tissues in the Logsdon and Segara datasets from the Oncomine database. B, Examples of immunohistochemistry (IHC) staining of *TOB1*. (B.1 and B.2) Pancreatic ductal adenocarcinoma (PDAC) tissues with negative and weak cytoplasmic *TOB1* staining; (B.3 and B.4) noncancerous tissues with moderate and strong cytoplasmic *TOB1* staining. C, Low levels of *TOB1* protein were observed in PDAC tissues by IHC. D, *TOB1* protein expression was negatively correlated with tumor size. E, Correlation between *TOB1* protein expression and overall survival in pancreatic cancer patients

To clarify the expression of *TOB1* in pancreatic cancer, we explored the mRNA and protein expression of *TOB1* in pancreatic samples via the Oncomine database and immunohistochemistry (IHC). Compared with normal pancreatic tissues, pancreatic cancer tissues showed significantly reduced *TOB1* mRNA (Figure 1A); the detailed data are shown in Table S1. By IHC, *TOB1* was shown to be mainly expressed in the cytoplasm (Figure 1B), and decreased *TOB1* protein levels were observed in PDAC tissues (Figure 1C).

To evaluate the clinical significance of *TOB1* in pancreatic cancer, we analyzed the association between *TOB1* expression and patients' clinicopathological characteristics (Table 1). Notably, *TOB1* protein expression was significantly correlated with tumor size (Figure 1D), suggesting that *TOB1* may play a role in negatively regulating the growth or proliferation of pancreatic cancer cells.

**Table 1:** Association between *TOB1* and the characteristics of pancreatic cancer patients

Clinical data	n	*TOB1* protein expression				*P*‐value
		−	+	++	+++	
Totally	108	44	56	6	2	
Gender						
Male	69	29	36	4	0	.546^a^
Female	39	15	20	2	2	
Age (years)						
≤60	52	25	27	0	0	**.030** a, *
>60	56	19	29	6	2	
Tumor *d*_max_						
≤5 cm	81	27	46	6	2	**.004** a, **
>5 cm	27	17	10	0	0	
Grade						
I	11	2	9	0	0	.311^b^
II	86	36	42	6	2	
III	11	6	5	0	0	
T						
T1 + T2	89	33	49	5	2	.109^a^
T3	19	11	7	1	0	
N						
N0	66	28	34	2	2	.571^a^
N1	42	16	22	4	0	
M						
M0	106	43	55	6	2	.765^a^
M1	2	1	1	0	0	
TNM stage						
I	50	19	28	1	2	.724^a^
II + IV	58	25	28	5	0	

Bold highlights statistically significant data.

a Mann‐Whitney U test was used for comparison between two groups.

b Kruskal‐Wallis H test was used for comparison of more than two groups.

* *P* < .05;

** *P* < .01.

We next analyzed the association between *TOB1* protein expression levels and survival time in 97 pancreatic cancer patients. Univariate analysis demonstrated that N stage and TNM stage were associated with the OS of pancreatic cancer patients in the Kaplan‐Meier analysis (Table S2). Patients with negative *TOB1* expression had worse outcomes than patients with positive *TOB1* expression (Figure 1E), although this finding was not significant.

We also analyzed the data in The Cancer Genome Atlas‐Pancreatic adenocarcinoma (TCGA‐PAAD), and found that *TOB1* was higher in cancer tissues (*t* = −2.208, *P* = .029, Table S3). After dividing *TOB1* mRNA expression into high and low according to the median, we analyzed the association between *TOB1* mRNA expression levels and survival time in 174 pancreatic cancer patients (four patients were excluded because of no survival data), which showed that patients with high *TOB1* mRNA expression had worse outcomes than patients with low *TOB1* mRNA expression (*P* = .002, Figure S2), but its mRNA expression level had no relationship with clinicopathological characteristics of PAAD tissues (Table S4).

### 3.2 *TOB1* overexpression reduces proliferation of human pancreatic cancer cells

**Figure 2:**
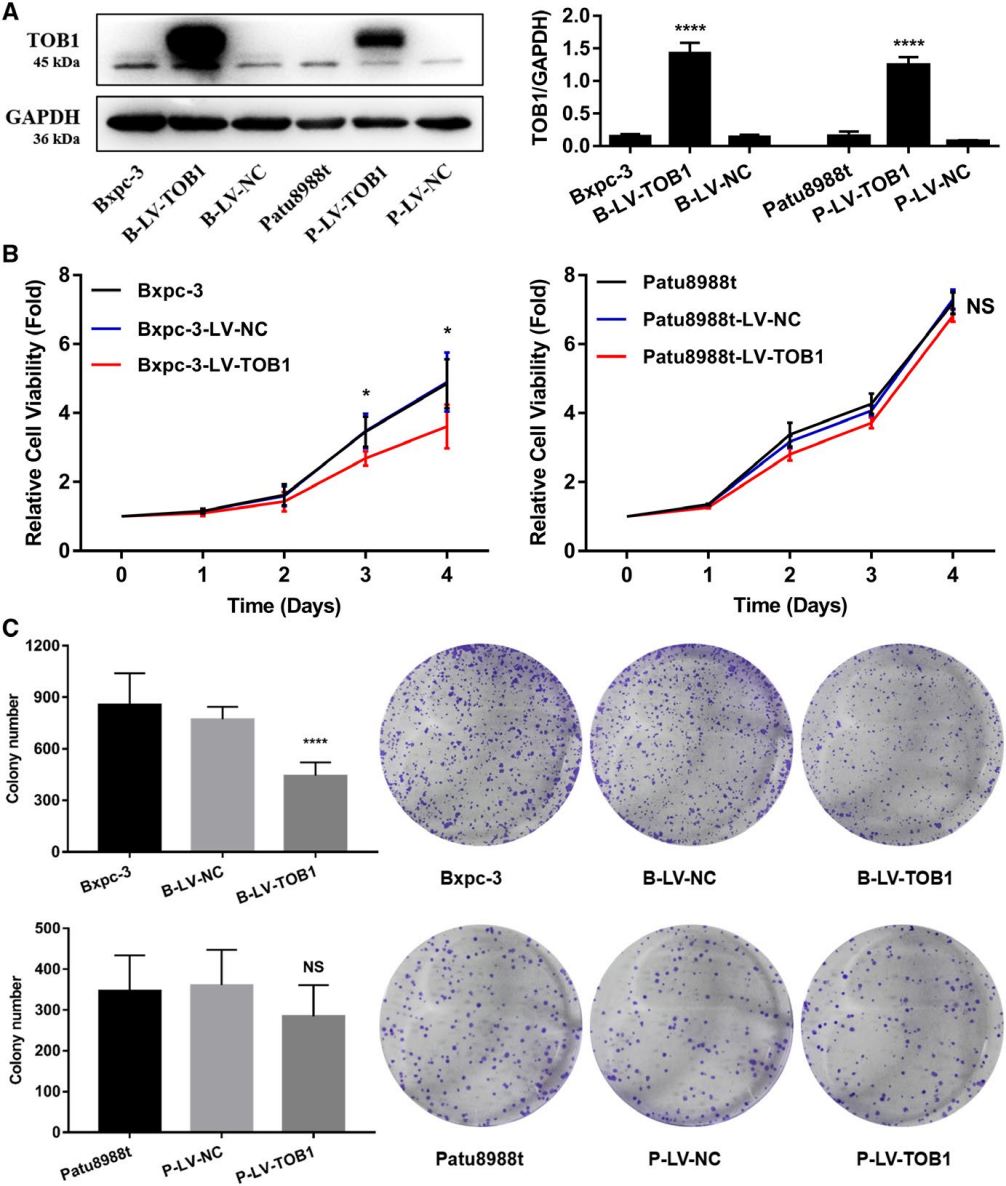
*TOB1* overexpression reduces proliferation and colony formation of Bxpc‐3 cells. A, *TOB1* protein was significantly increased in *TOB1* stable transfectants compared with the corresponding NC and parental cells. B, Overexpressing *TOB1* inhibited the cell viability of Bxpc‐3 cells. C, Ectopic expression of *TOB1* remarkably reduced the size and number of clones in Bxpc‐3 cells. B‐LV‐NC, BxPC‐3‐LV‐NC; B‐LV‐T, BxPC‐3‐LV‐*TOB1*; P‐LV‐NC, Patu8988t‐LV‐NC; P‐LV‐T, Patu8988t‐LV‐*TOB1*. Error bars indicate the mean ± SD. NS, no significance. *P < .05; ****P < .0001

Given that *TOB1* is involved in regulating the biological behaviors of various tumors, we examined whether *TOB1* acts as a tumor suppressor in pancreatic cancer cells by utilizing a gain‐of‐function approach. BxPC‐3‐LV‐*TOB1* and Patu8988t‐LV‐*TOB1* stable transfectants were established using lentivirus containing *TOB1* cDNA. *TOB1* stable transfectants were selected and confirmed by WB. As shown in Figure 2A, *TOB1* was highly expressed in *TOB1* stable transfectants compared with the corresponding NC and parental cells. In addition, the fluorescence intensity of *TOB1*‐overexpressing cells was stronger than that in the corresponding NC and parental cells, and *TOB1* protein was mainly expressed in the cytoplasm (Figure S3), consistent with the results of IHC.

**Figure 3:**
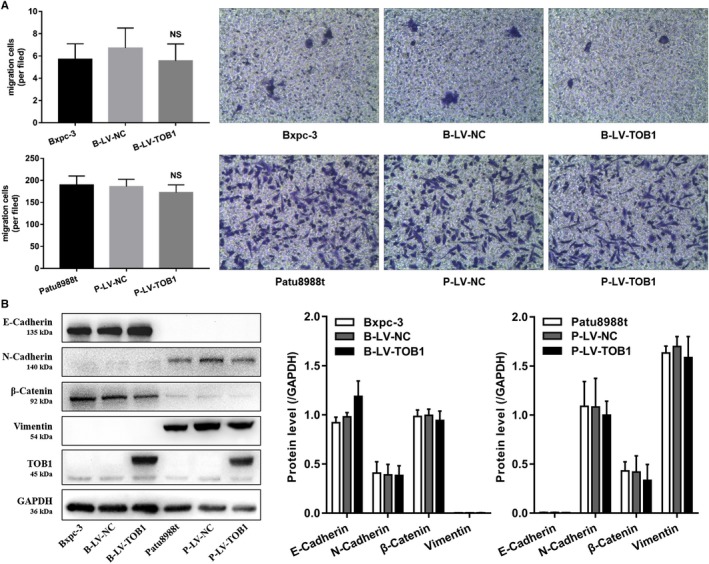
*TOB1* overexpression has no impact on cell migration and expression of EMT‐related proteins. A, Overexpression of *TOB1* had no impact on the migration of pancreatic cancer cells. B, No obvious change in the expression of EMT‐related markers occurred after overexpressing *TOB1*. B‐LV‐NC, BxPC‐3‐LV‐NC; B‐LV‐T, BxPC‐3‐LV‐*TOB1*; P‐LV‐NC, Patu8988t‐LV‐NC; P‐LV‐T, Patu8988t‐LV‐*TOB1*. Error bars indicate the mean ± SD. NS, no significance

*TOB1* overexpression inhibited cell viability (Figure 2B) and remarkably reduced the size and number of clones (Figure 2C). Interestingly, these results occurred in BxPC‐3 cells (*K‐Ras* wild‐type) but not in Patu8988t cells (*K‐Ras* mutated). Metastasis is one of the toughest challenges in PDAC, and epithelial‐to‐mesenchymal transformation (EMT) is critical to the metastasis cascade of epithelial‐derived solid tumor. We also assessed whether *TOB1* affects migration‐ or EMT‐related invasion. A transwell migration assay found that overexpression of *TOB1* had no significant effect on cell migration in these two cell lines (Figure 3A). The expression of several EMT‐related markers, such as E‐cadherin, N‐cadherin, beta‐catenin, and Vimentin, was evaluated by WB. As shown in Figure 3B, E‐cadherin was slightly increased and N‐cadherin, beta‐catenin, and Vimentin were slightly decreased after overexpressing *TOB1*, but there was no statistically significant difference. Taken together, these results indicate that the mechanism of *TOB1* against pancreatic cancer might be an antiproliferative effect rather than inhibition of invasion or metastasis in BxPC‐3 cells in vitro.

### 3.3 *TOB1* mediates the antiproliferative effect through alterations in the cell cycle

**Figure 4:**
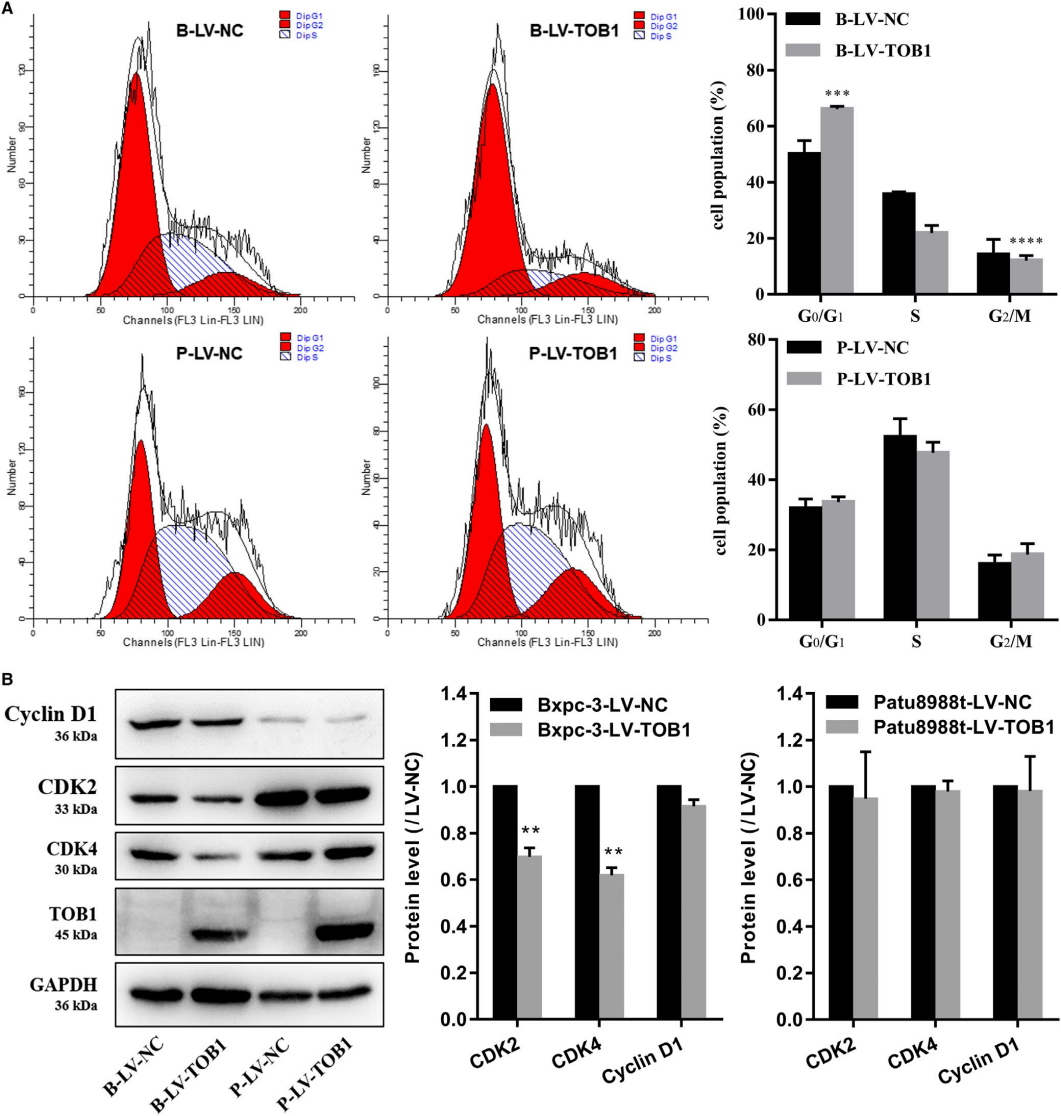
Overexpression of *TOB1* promotes cell cycle arrest in Bxpc‐3 cells. A, FACS analysis of the effect of *TOB1* on cell cycle analysis. B, Cell cycle proteins were evaluated by Western blot. B‐LV‐NC, BxPC‐3‐LV‐NC; B‐LV‐*TOB1*, BxPC‐3‐LV‐*TOB1*; P, P‐LV‐NC, Patu8988t‐LV‐NC; P‐LV‐*TOB1*, Patu8988t‐LV‐*TOB1*. Error bars indicate the mean ± SD. **P < .01; ***P < .001; ****P < .0001

Cell cycle analysis showed that overexpression of *TOB1* in Bxpc‐3 cells significantly blocked the transition from G_1_ to S phase compared with negative control cells, but there was no significant difference in Patu8988t cells (Figure 4A).

Based on the above results, we studied the effect of *TOB1* expression on cell cycle modulators. We evaluated alterations in *CDK2*, *CDK4*, and cyclin D1 levels in *TOB1*‐overexpressing cells compared to the negative control cells by WB. Figure 4B shows that cyclin D1 expression was slightly decreased in BxPC‐3‐LV‐*TOB1* cells, and *CDK2* and *CDK4* were strongly downregulated after overexpression of *TOB1* in Bxpc‐3 cells. Thus, the antiproliferative effects of *TOB1* are likely mediated by regulating cell cycle progression through *CDK2* and *CDK4*.

### 3.4 *Foxa2* regulates *TOB1* expression, and *TOB1* targets the calcium pathway genes

After observation of the reversal of the malignant phenotype of pancreatic cancer cells by overexpressing *TOB1*, we explored the underlying molecular mechanisms. Specifically, the mechanistic research involved analysis of the upstream transcription factors and the downstream signaling pathways.

**Figure 5:**
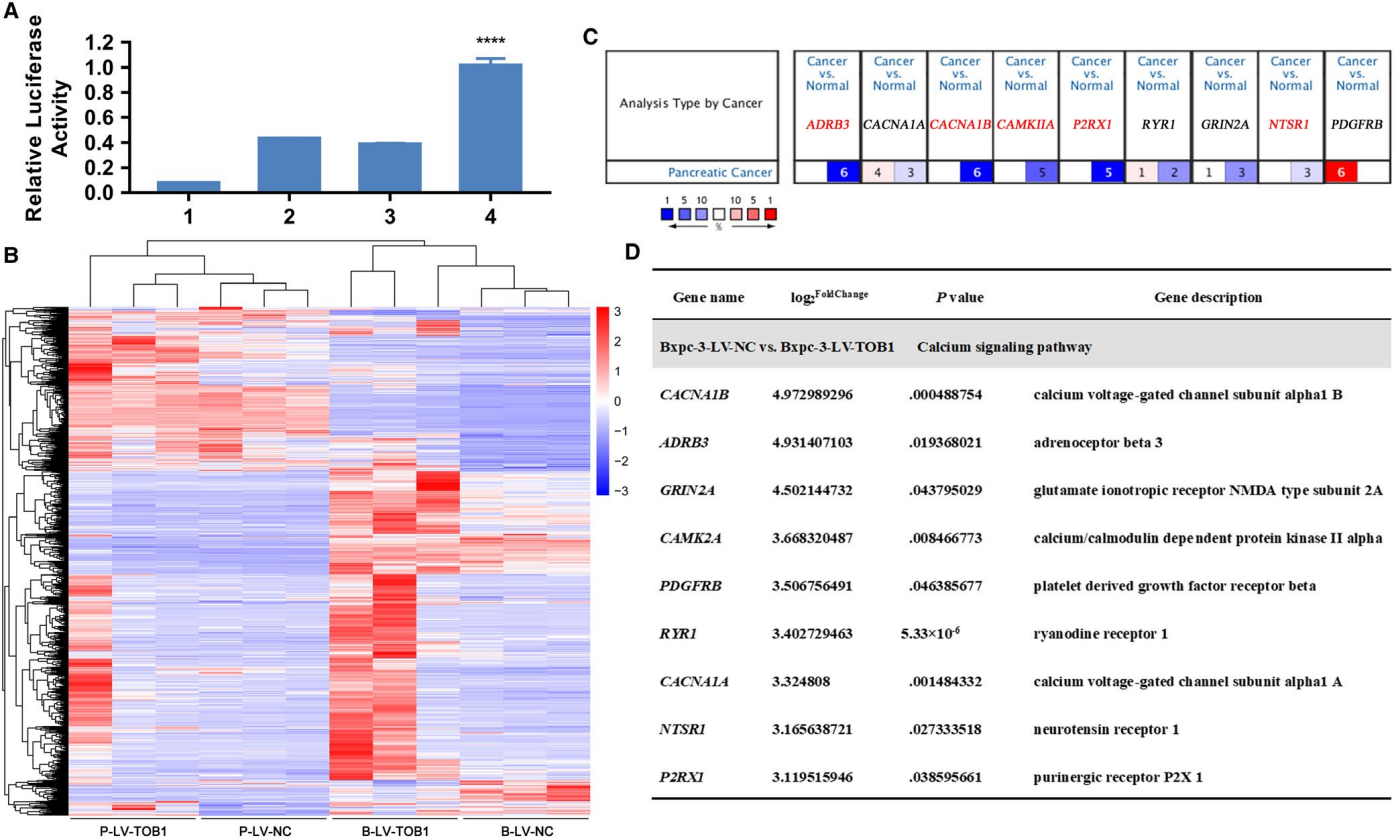
*TOB1* regulates the expression of calcium pathway genes in pancreatic cancer. A, Luciferase reporter assay. 1, Promoter‐NC + TF‐NC; 2, Promoter‐NC + *Foxa2*; 3, *TOB1* Promoter + TF‐NC; 4, *TOB1* Promoter + *Foxa2*. B, All cluster heatmaps of differential genes. C, mRNA expression of some typical differentially expressed genes involved in the calcium pathway in pancreatic cancer tissues. The number in cells represents the number of analyses meeting the thresholds (*P*‐value < .05, fold change = all, gene rank = all; data type: mRNA). Gene rank determines cell color. Specifically, a stronger red or blue color indicates a more pronounced upregulation or downregulation of the gene. D, Some typical differentially expressed genes involved in the calcium pathway after overexpressing *TOB1*. B‐LV‐NC, BxPC‐3‐LV‐NC; B‐LV‐*TOB1*, BxPC‐3‐LV‐*TOB1*; P‐LV‐NC, Patu8988t‐LV‐NC; P‐LV‐*TOB1*, Patu8988t‐LV‐*TOB1*. **** *P* ＜ .0001

We first used TFBIND software to search for transcription factor binding sites of *TOB1* (data not shown). Among transcription factors with a score greater than 0.90, *Foxa2*, which showed decreased expression and related to the malignant phenotype of pancreatic cancer cells, was selected for further analysis.^20^, ^21^, ^22^ As shown in Figure 5A, a luciferase reporter assay revealed that overexpression of *Foxa2* significantly enhanced *TOB1* promoter activity, indicating that *Foxa2* may act upstream of *TOB1* and regulate its mRNA expression.

To identify related genes that are regulated by *TOB1* in pancreatic cancer, we performed RNA‐Seq analyses of controls or transfectants. The data revealed that 795 differential mRNAs were detected in the BxPC‐3‐LV‐*TOB1* vs BxPC‐3‐LV‐NC groups (Figure 5B). Through KEGG (Kyoto Encyclopedia of Genes and Genomes) pathway analysis, 56 pathways were found to be involved in the BxPC‐3‐LV‐*TOB1* vs BxPC‐3‐LV‐NC groups (Table S5), and it is worth noting that the calcium pathway showed significant enrichment. However, few differential genes and pathways were found in the Patu8988t‐LV‐*TOB1* vs Patu8988t‐LV‐NC groups (Table S6). From these data, we found that after overexpression of *TOB1*, more genes and pathways are involved in the biological behavior of *K‐Ras* wild‐type cells than *K‐Ras* mutated cells, consistent with the results of cytological phenotype.

To assess the expression of some typical differentially expressed genes involved in the calcium pathway in pancreatic cancer tissues, we performed an analysis via the Oncomine database and determined that some of these genes, such as *CACNA1B*, *ADRB3*, *P2RX1*, *CAMKIIA*, and *NTSR1*, had lower expression in the cancer tissues than normal tissues (Figure 5C), while these genes were elevated after *TOB1* overexpression (Figure 5D). These findings indirectly suggest that *TOB1* is likely to function through these calcium pathway genes.

In summary, *Foxa2*, as a transcription factor of *TOB1*, regulates its mRNA expression, while *TOB1* participates in the anti‐pancreatic cancer process by regulating the expression of calcium pathway genes.

## 4 DISCUSSION

During previous decades, growing evidence has emphasized the rising importance of *TOB1* in diversified human cancers, but how *TOB1* works in pancreatic cancer has not been fully expounded. Therefore, we tried to explore whether altering *TOB1* expression could affect the development of pancreatic cancer and elucidate its potential mechanism.

Originally, Yoshida^9^ discovered that *TOB1* knockout mice spontaneously formed tumors and suggested that the *TOB1* gene played an important role in the formation of malignant tumors. Hereafter, abnormal *TOB1* expression has been reported in many human malignant tumors, including breast cancer,^11^ lung cancer,^10^ papillary thyroid cancer,^12^ skin squamous cell carcinoma, 23 and gastric cancer.^24^ Studies also showed that the *TOB1* expression level was correlated with the clinicopathological characteristics of tumors and could be used as a prognostic marker for some tumors. Among breast cancer patients without lymph node metastasis, patients with lower *TOB1* expression had a worse prognosis.^18^ In patients with intestinal gastric cancer, low *TOB1* and high phosphorylation of *TOB1* both predicted poor prognosis.^25^ No studies have been conducted on the expression of *TOB1* in pancreatic cancer tissues.

We discussed *TOB1* expression and its clinical significance in pancreatic cancer tissues. Compared with normal pancreatic tissues, pancreatic cancer tissues showed substantially decreased *TOB1* mRNA and protein levels. In addition, by analyzing the correlation between *TOB1* protein and the clinicopathological features of pancreatic cancer patients, we found that larger cancer tissues had a lower *TOB1* protein level. Although there was no significant difference, patients with negative *TOB1* expression had worse outcomes. This result may be explained by the limited number of cases, and increased sample sizes are needed to confirm the prognostic value of this molecule in pancreatic cancer patients. But, we got an opposite result in TCGA‐PAAD analysis. As we all know, ductal adenocarcinoma is an invasive mucin‐producing and duct‐forming epithelial neoplasm with an intense stromal desmoplastic response, often presents low neoplastic cellularity, only 5%‐20%.^26^ Prior genome sequencing studies employed techniques to purify tumor samples, which makes that samples with low neoplastic cellularity have been underrepresented, even though low cellularity cancers comprise the majority of surgically resected PDAC.^27^ Besides, there are only four normal control cases in TCGA‐PAAD. Given this, we considered that the analysis result of TCGA‐PAAD may be not very reliable. To sum up, we concluded that *TOB1* expression is downregulated in pancreatic cancer and may act as a tumor suppressor.

As a candidate tumor suppressor gene, *TOB1* is also involved in regulating proliferation, apoptosis, invasion, migration, and radiochemotherapeutic sensitivity of various tumor cells.^15^, ^17^, ^18^, ^28^, ^29^, ^30^ We demonstrated that overexpression of *TOB1* could inhibit proliferation and colony formation. The cell cycle analysis suggested that *TOB1* overexpression led to cell aggregation in G_1_ phase and cell depletion in S phase by decreasing the expression of *CDK2* and *CDK4*. A strong relationship between *TOB1* and cyclin D1 has been reported in the literature,^31^, ^32^, ^33^ but we did not observe a correlation between *TOB1* expression and cyclin D1. No effects on cell migration or expression of EMT‐related markers were found in our study. All the above results demonstrated that the antitumor effect of *TOB1* in *K‐Ras* wild‐type pancreatic cancer cells is due to its antiproliferative effect. Abnormal activation of *K‐Ras* is present in more than 90% of PDAC,^34^ which represents a molecular typing of pancreatic cancer. One study found that *TOB1* was involved in the regulation of chemotherapeutic sensitivity in HER2‐positive breast cancers,^18^ suggesting that *TOB1* plays different roles in different molecular subtypes of cancer. From our results, we found that *TOB1* may function as a tumor suppressor gene in *K‐Ras* wild‐type pancreatic cancer and dysregulated *TOB1* may partially interpret the underlying mechanism of *K‐Ras* wild‐type pancreatic cancer carcinogenesis, but what roles it plays in *K‐Ras* mutant pancreatic cancer cells deserve further study.

Some studies have suggested that the cytoplasmic localization of *TOB1* is required for its antiproliferative effect.^14^ In contrast, another study reported that the nuclear localization of *TOB1* had antiproliferative activity.^13^ Prior studies have also noted the importance of phosphorylation of *TOB1*.^10^, ^12^ Our tissue and cell studies demonstrated that in pancreatic cancer, *TOB1* protein was mainly located in the cytoplasm, indicating that its antiproliferative effect may be independent of nuclear localization.

Several reports have shown that *TOB1* can function through a variety of pathways, including Smad 4/β‐catenin,^28^ PI3K/PTEN,^15^ and MAPK/ERK.^29^ Similarly, in our analysis of pathways, some typical pathways were included, such as the MAPK and TNF signaling pathways. Beyond that, the most obvious finding to emerge from pathway analysis is that *TOB1* likely works through regulating the calcium pathway genes. Ca^2+^ is a ubiquitous cell signaling molecule for various physiological and pathological processes.^35^, ^36^ In the 1970s, researchers discovered that cell proliferation may be regulated by calcium pathways,^37^ and some key steps in cell cycle progression are dependent on calcium signaling, especially the early transition into the G_1_, G_1_/S, and G_2_/M phases.^38^ Prior studies have also noted the importance of calcium signaling and tumors.^39^, ^40^, ^41^, ^42^, ^43^, ^44^, ^45^, ^46^, ^47^ Davis^48^ found that the concentration of Ca^2+^ in the cytoplasm was temporarily increased when using epidermal growth factor (EGF) and hypoxia to induce EMT. Conversely, EGF and hypoxia‐induced EMT were reduced when decreasing the calcium in breast cancer cells.^48^ Giorgi^49^ proposed that *TP53* mutation could inhibit cell apoptosis through the calcium signaling pathway. Tumors driven by *H‐Ras* were reported to be maintained by calcium signaling pathways.^50^ Wang^51^ established that the Wnt‐Ca^2+^ pathway was a key pathway that promoted the development and progression of tumors with *K‐Ras* mutations and proposed that blocking the combination of *K‐Ras* and calmodulin could be a potential treatment strategy for cancer with *K‐Ras* mutations. It is important to determine whether the *TOB1* calcium pathways could be used as therapeutic targets in the further study.

In summary, our study demonstrates that *TOB1* expression is downregulated in human pancreatic cancer. Overexpression of *TOB1* can partially reverse the malignant phenotype of *K‐Ras* wild‐type pancreatic cancer cells by regulating calcium pathway genes.

## CONFLICT OF INTEREST

No conflict of interest.

## AUTHOR CONTRIBUTIONS

Conceptualization, Yuru Bai, Lu Qiao, Ning Xie, Yan Li, Yongzhan Nie, Yan Pan, Yupeng Shi, Jinhai Wang, and Na Liu; Data curation, Yuru Bai and Lu Qiao; Formal analysis, Yuru Bai; Funding acquisition, Jinhai Wang and Na Liu; Investigation, Yuru Bai; Methodology, Yuru Bai, Lu Qiao and Yan Li; Project administration, Jinhai Wang and Na Liu; Resources, Yuru Bai and Na Liu; Software, Yuru Bai and Lu Qiao; Supervision, Na Liu; Validation, Yuru Bai, Lu Qiao and Yan Li; Visualization, Yuru Bai and Na Liu; Writing—original draft, Yuru Bai; Writing—review & editing, Yuru Bai and Na Liu.
