# Supplementary figures and images for: TOB1 suppresses proliferation in K‐Ras wild‐type pancreatic cancer
Source: Cancer Med. 2019 Dec 31;9(4):1503–14. doi: 10.1002/cam4.2756 (PMC7013073; doi:10.1002/cam4.2756)

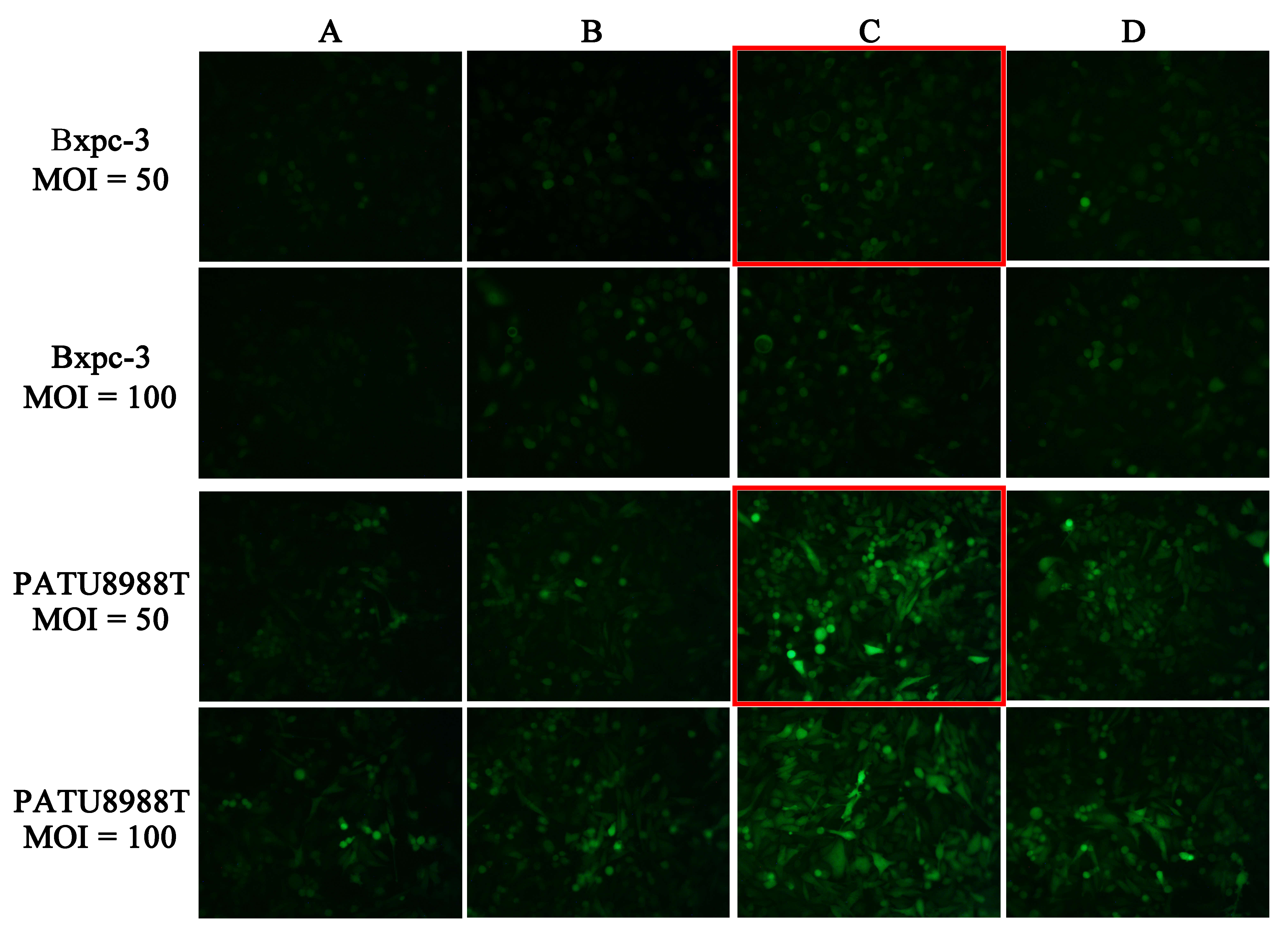

Supplement: Supplementary file 1 [file CAM4-9-1503-s001.tif]

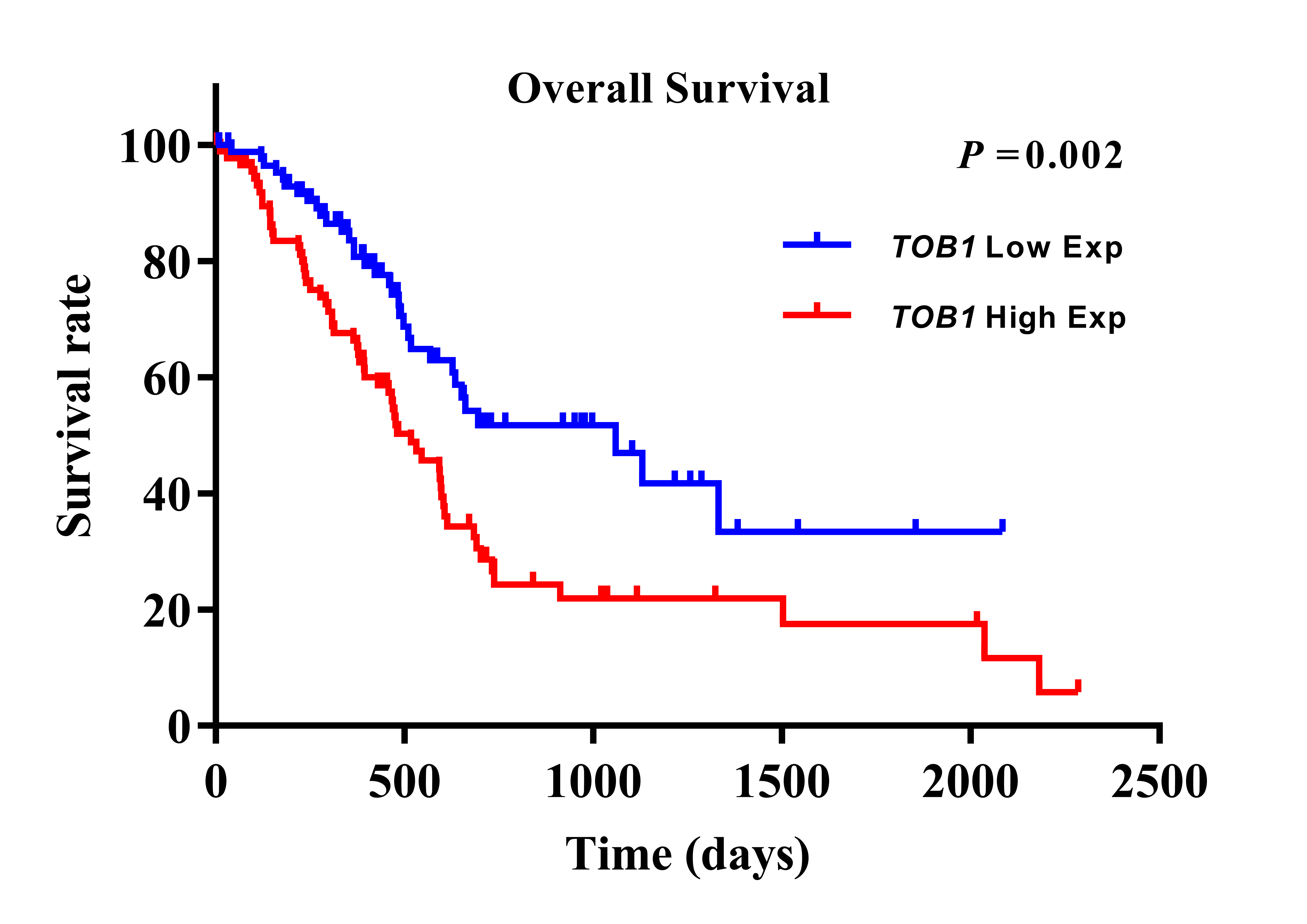

Supplement: Supplementary file 2 [file CAM4-9-1503-s002.tif]

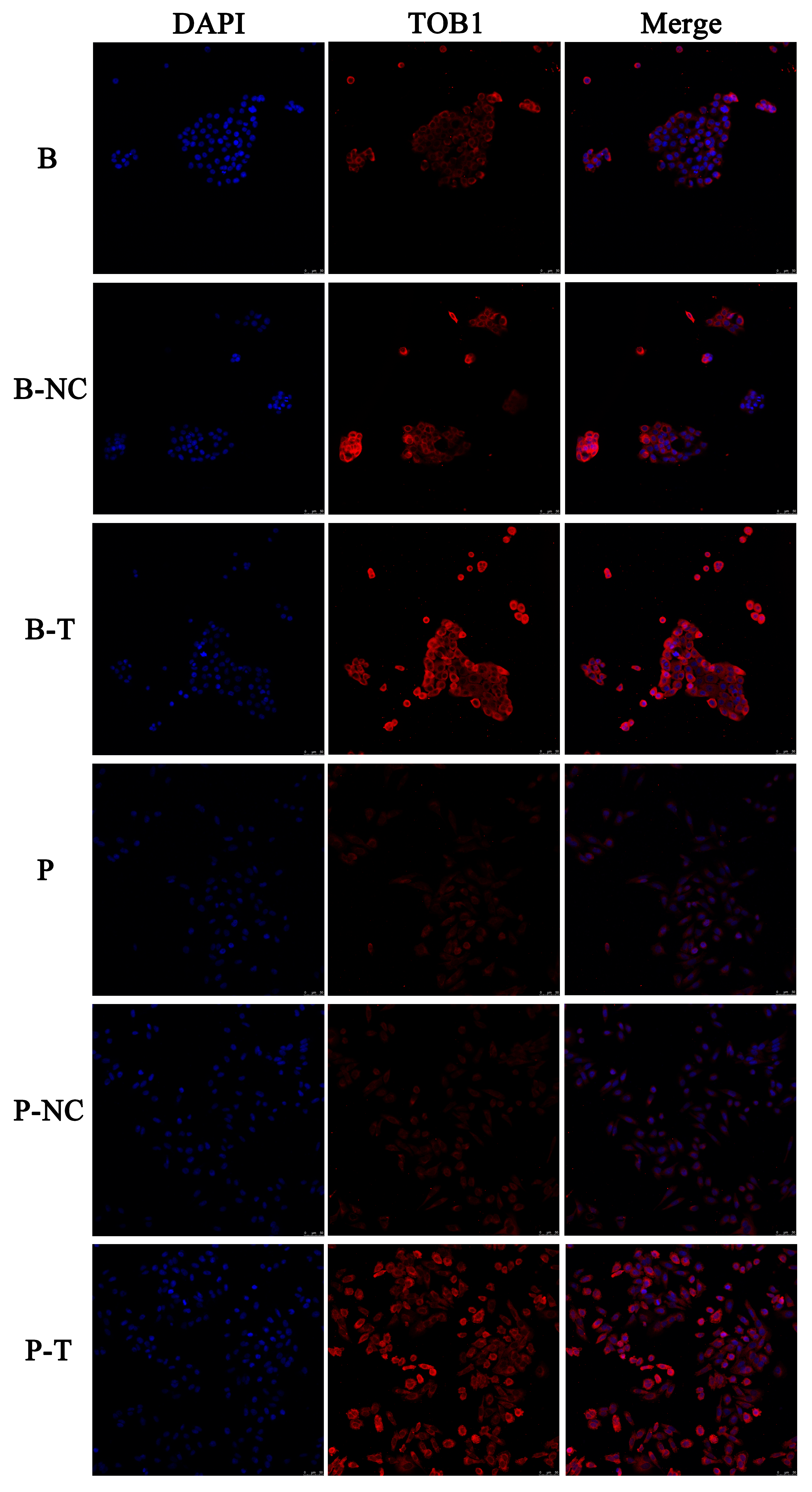

Supplement: Supplementary file 3 [file CAM4-9-1503-s003.tif]
