# Supplementary material for: TOB1 suppresses proliferation in K‐Ras wild‐type pancreatic cancer
Source: Cancer Med. 2019 Dec 31;9(4):1503–14. doi: 10.1002/cam4.2756 (PMC7013073; doi:10.1002/cam4.2756)
Supplement: Supplementary file 4 [file CAM4-9-1503-s004.doc]

Table S1 mRNA expression of TOB1 in human pancreas tissues

| *Gene* | *Dataset* | *Normal (Cases)* | *Tumor (Cases)* | *Fold change* | *t-Test* | *P-value* |
| --- | --- | --- | --- | --- | --- | --- |
| *TOB1* | Logsdon | Pancreas (5) | Pancreatic Adenocarcinoma (10) | -2.238 | -2.851 | 0.008 |
|  | Segara | Pancreas (6) | Pancreatic Carcinoma (11) | -3.157 | -2.543 | 0.011 |
