# Supplementary material for: TOB1 suppresses proliferation in K‐Ras wild‐type pancreatic cancer
Source: Cancer Med. 2019 Dec 31;9(4):1503–14. doi: 10.1002/cam4.2756 (PMC7013073; doi:10.1002/cam4.2756)
Supplement: Supplementary file 5 [file CAM4-9-1503-s005.doc]

Table S2 Kaplan-Meier analyses of overall survival in pancreatic cancer patients (n=97)

| Variables | n | *χ^2^* | *P* |
| --- | --- | --- | --- |
| Gender |  |  |  |
| Female | 36 | 0.656 | 0.418 |
| Male | 61 |
| Age (years) |  |  |  |
| ≤ 60 | 47 | 0.788 | 0.375 |
| > 60 | 50 |
| Tumor d_max_ |  |  |  |
| ≤ 5 cm | 72 | 1.126 | 0.289 |
| > 5 cm | 25 |
| G |  |  |  |
| G1 | 11 | 3.429 | 0.18 |
| G2 | 76 |
| G3 | 10 |
| T |  |  |  |
| T1+T2 | 78 | 0.108 | 0.742 |
| T3 | 19 |
| **N** |  |  |  |
| N0 | 59 | 10.263 | **0.001** |
| N1 | 38 |
| M |  |  |  |
| M0 | 95 | 0.781 | 0.377 |
| M1 | 2 |
| **TNM stage** |  |  |  |
| Ⅰ | 43 | 9.403 | **0.002** |
| Ⅱ + Ⅳ | 54 |
| TOB1 Expression |  |  |  |
| negative | 39 | 1.6 | 0.206 |
| positive | 58 |
