# Supplementary material for: TOB1 suppresses proliferation in K‐Ras wild‐type pancreatic cancer
Source: Cancer Med. 2019 Dec 31;9(4):1503–14. doi: 10.1002/cam4.2756 (PMC7013073; doi:10.1002/cam4.2756)
Supplement: Supplementary file 6 [file CAM4-9-1503-s006.doc]

**Table S3 mRNA expression of *TOB1* in human pancreas tissues**

**(data form TCGA-PAAD)**

| group | n | Mean of *TOB1* mRNA Expression | *t*-test | *p* |
| --- | --- | --- | --- | --- |
| Normal tissues | 4 | 2023.00 | -2.208 | **0.029** |
| Cancer tissues | 178 | 5579.25 |
