# Supplementary material for: TOB1 suppresses proliferation in K‐Ras wild‐type pancreatic cancer
Source: Cancer Med. 2019 Dec 31;9(4):1503–14. doi: 10.1002/cam4.2756 (PMC7013073; doi:10.1002/cam4.2756)
Supplement: Supplementary file 7 [file CAM4-9-1503-s007.doc]

**Table S4 Association between *TOB1*** mRNA and clinicopathological characteristics of TCGA-PAAD tissues

| **Clinical Data** | **n** | ***TOB1* mRNA Expression** | | ***χ^2^*** | ***P*** |
| --- | --- | --- | --- | --- | --- |
|  |  | **low** | **high** |  |  |
| Totally | 175 | 87 | 88 |  |  |
| Gender |  |  |  |  |  |
| Female | 80 | 36 | 44 | 1.31 | 0.252 |
| Male | 95 | 51 | 44 |  |  |
| Age |  |  |  |  |  |
| ＜60 | 50 | 26 | 24 | 0.146 | 0.702 |
| ≥60 | 125 | 61 | 64 |  |  |
| T |  |  |  |  |  |
| T1+T2 | 30 | 18 | 12 | 1.532 | 0.216 |
| T3+T4 | 145 | 69 | 76 |  |  |
| N |  |  |  |  |  |
| N0 | 52 | 28 | 24 | 0.505 | 0.477 |
| N1 | 123 | 59 | 64 |  |  |
| M |  |  |  |  |  |
| M0 | 170 | 86 | 84 | 1.818 | 0.368 |
| M1 | 5 | 1 | 4 |  |  |
| TNM stage |  |  |  |  |  |
| Ⅰ+Ⅱ | 166 | 83 | 83 | 0.105 | 1.000 |
| Ⅲ+Ⅳ | 9 | 4 | 5 |  |  |
