# Supplementary material for: TOB1 suppresses proliferation in K‐Ras wild‐type pancreatic cancer
Source: Cancer Med. 2019 Dec 31;9(4):1503–14. doi: 10.1002/cam4.2756 (PMC7013073; doi:10.1002/cam4.2756)
Supplement: Supplementary file 8 [file CAM4-9-1503-s008.doc]

**Table S5** the KEGG pathway analysis in the BxPC-3-LV-TOB1 vs BxPC-3-LV-NC group

| ID | Description | Gene Ratio | *P* value | Count |
| --- | --- | --- | --- | --- |
| hsa05146 | Amoebiasis | 15/282 | 7.12E-06 | 15 |
| **hsa04020** | **Calcium signaling pathway** | **22/282** | **8.52E-06** | **22** |
| hsa04725 | Cholinergic synapse | 16/282 | 1.76E-05 | 16 |
| hsa04724 | Glutamatergic synapse | 15/282 | 0.000119601 | 15 |
| hsa04720 | Long-term potentiation | 11/282 | 0.0001493 | 11 |
| hsa04657 | IL-17 signaling pathway | 12/282 | 0.000239767 | 12 |
| hsa05033 | Nicotine addiction | 7/282 | 0.000290287 | 7 |
| hsa04080 | Neuroactive ligand-receptor interaction | 21/282 | 0.000845735 | 21 |
| hsa04713 | Circadian entrainment | 12/282 | 0.001159124 | 12 |
| hsa04668 | TNF signaling pathway | 12/282 | 0.001660415 | 12 |
| hsa05031 | Amphetamine addiction | 9/282 | 0.001864467 | 9 |
| hsa04925 | Aldosterone synthesis and secretion | 10/282 | 0.002000162 | 10 |
| hsa04974 | Protein digestion and absorption | 10/282 | 0.002436045 | 10 |
| hsa04610 | Complement and coagulation cascades | 9/282 | 0.002591662 | 9 |
| hsa04921 | Oxytocin signaling pathway | 15/282 | 0.003134699 | 15 |
| hsa04261 | Adrenergic signaling in cardiomyocytes | 14/282 | 0.003520094 | 14 |
| hsa04024 | cAMP signaling pathway | 17/282 | 0.00366566 | 17 |
| hsa04010 | MAPK signaling pathway | 22/282 | 0.00735913 | 22 |
| hsa04060 | Cytokine-cytokine receptor interaction | 17/282 | 0.008422536 | 17 |
| hsa05332 | Graft-versus-host disease | 3/282 | 0.008564229 | 3 |
| hsa04072 | Phospholipase D signaling pathway | 13/282 | 0.008979222 | 13 |
| hsa04670 | Leukocyte transendothelial migration | 11/282 | 0.008992677 | 11 |
| hsa04728 | Dopaminergic synapse | 12/282 | 0.009658259 | 12 |
| hsa04015 | Rap1 signaling pathway | 17/282 | 0.010174194 | 17 |
| hsa04510 | Focal adhesion | 16/282 | 0.011909923 | 16 |
| hsa04916 | Melanogenesis | 10/282 | 0.0123307 | 10 |
| hsa00350 | Tyrosine metabolism | 5/282 | 0.013248797 | 5 |
| hsa00980 | Metabolism of xenobiotics by cytochrome P450 | 7/282 | 0.013375273 | 7 |
| hsa04730 | Long-term depression | 7/282 | 0.013375273 | 7 |
| hsa04950 | Maturity onset diabetes of the young | 4/282 | 0.018110375 | 4 |
| hsa04512 | ECM-receptor interaction | 8/282 | 0.018862942 | 8 |
| hsa04514 | Cell adhesion molecules (CAMs) | 10/282 | 0.019310168 | 10 |
| hsa05134 | Legionellosis | 6/282 | 0.01977486 | 6 |
| hsa04380 | Osteoclast differentiation | 10/282 | 0.020504633 | 10 |
| hsa05323 | Rheumatoid arthritis | 7/282 | 0.020583582 | 7 |
| hsa04911 | Insulin secretion | 8/282 | 0.021789828 | 8 |
| hsa04062 | Chemokine signaling pathway | 13/282 | 0.023122342 | 13 |
| hsa05204 | Chemical carcinogenesis | 7/282 | 0.024107076 | 7 |
| hsa05418 | Fluid shear stress and atherosclerosis | 11/282 | 0.02605349 | 11 |
| hsa00380 | Tryptophan metabolism | 5/282 | 0.026552019 | 5 |
| hsa04750 | Inflammatory mediator regulation of TRP channels | 9/282 | 0.026579149 | 9 |
| hsa00360 | Phenylalanine metabolism | 3/282 | 0.027491974 | 3 |
| hsa04742 | Taste transduction | 7/282 | 0.030137116 | 7 |
| hsa05133 | Pertussis | 7/282 | 0.03235405 | 7 |
| hsa05144 | Malaria | 5/282 | 0.032379358 | 5 |
| hsa04940 | Type I diabetes mellitus | 3/282 | 0.032736061 | 3 |
| hsa05140 | Leishmaniasis | 6/282 | 0.033316038 | 6 |
| hsa04611 | Platelet activation | 10/282 | 0.037409842 | 10 |
| hsa04022 | cGMP-PKG signaling pathway | 12/282 | 0.039045827 | 12 |
| hsa04971 | Gastric acid secretion | 7/282 | 0.039651048 | 7 |
| hsa05321 | Inflammatory bowel disease (IBD) | 5/282 | 0.042514194 | 5 |
| hsa05202 | Transcriptional misregulation in cancer | 13/282 | 0.044098377 | 13 |
| hsa04726 | Serotonergic synapse | 9/282 | 0.046249073 | 9 |
| hsa04640 | Hematopoietic cell lineage | 7/282 | 0.047948904 | 7 |
| hsa05200 | Pathways in cancer | 30/282 | 0.048141226 | 30 |
| hsa04924 | Renin secretion | 6/282 | 0.048338879 | 6 |

ID: unique number information of pathway in KEGG database; Description: the description of the pathway; Gene Ratio: the ratio of the number of differential genes involved in the pathway to the number of all differential genes; P value: statistical difference level; gene ID: ID of differential genes to the pathway; Count: the number of differential genes related to the pathway.
