# Supplementary material for: TOB1 suppresses proliferation in K‐Ras wild‐type pancreatic cancer
Source: Cancer Med. 2019 Dec 31;9(4):1503–14. doi: 10.1002/cam4.2756 (PMC7013073; doi:10.1002/cam4.2756)
Supplement: Supplementary file 9 [file CAM4-9-1503-s009.doc]

**Table S6** the KEGG pathway analysis in the Patu8988t-LV-TOB1 vs Patu8988t-LV-NC group

| ID | Description | Gene Ratio | *P* value | Count |
| --- | --- | --- | --- | --- |
| hsa04020 | Calcium signaling pathway | 11/78 | 8.23304E-06 | 11 |
| hsa04921 | Oxytocin signaling pathway | 7/78 | 0.002664053 | 7 |
| hsa04060 | Cytokine-cytokine receptor interaction | 7/78 | 0.009311315 | 7 |
| hsa04371 | Apelin signaling pathway | 5/78 | 0.026140756 | 5 |
| hsa04022 | cGMP-PKG signaling pathway | 5/78 | 0.046308712 | 5 |
| hsa04730 | Long-term depression | 4/78 | 0.004911775 | 4 |
| hsa04924 | Renin secretion | 4/78 | 0.005595322 | 4 |
| hsa04970 | Salivary secretion | 4/78 | 0.015421737 | 4 |
| hsa04512 | ECM-receptor interaction | 4/78 | 0.016844331 | 4 |
| hsa04974 | Protein digestion and absorption | 4/78 | 0.019930339 | 4 |
| hsa05146 | Amoebiasis | 4/78 | 0.023345043 | 4 |
| hsa00220 | Arginine biosynthesis | 2/78 | 0.029252529 | 2 |

ID: unique number information of pathway in KEGG database; Description: the description of the pathway; Gene Ratio: the ratio of the number of differential genes involved in the pathway to the number of all differential genes; P value: statistical difference level; gene ID: ID of differential genes to the pathway; Count: the number of differential genes related to the pathway.
