# Supplementary material for: TOB1 suppresses proliferation in K‐Ras wild‐type pancreatic cancer
Source: Cancer Med. 2019 Dec 31;9(4):1503–14. doi: 10.1002/cam4.2756 (PMC7013073; doi:10.1002/cam4.2756)
Supplement: Supplementary file 10 [file CAM4-9-1503-s010.pdf]

中国典型培养物保藏中心

CHINA CENTER FOR TYPE CULTURE COLLECTION (CCTCC)

Wuhan University, Wuhan 430072, China

Phone: 86-027-68752093 **Fax:** 86-027-68754833 **Email:** shenchao@whu.edu.cn

---

3-2-2017

Entrusted by the Second Affiliated Hospital of Xi'an Jiaotong University, CCTCC has conducted identification experiments on the Patu8988 cell line, and come to the following conclusions:

1. There was no third allele found in Patu8988 cell line, it indicating that there was no cross-contaminant of human source cell line.
2. Compared the STR data of Patu8988 cell line in the databases of ATCC and DSMZ, all the locations of Patu8988 were exactly matched with the locations of PA-TU-8988T cells found in DSMZ cell bank, so it is PA-TU-8988T cell line (Table 1).

Manager:

China Center for Type Culture Collection

Table 1. The alleles of 21 locations in Patu8988 cell line

Patu8988 cell line（Fig. No.XB5259）		
Marker	Allele 1	Allele 2
D19S433	14	14
D5S818	11	13
D21S11	26	26
D18S51	12	12
D6S1043	10	13
AMEL	X	X
D3S1358	15	15
D13S317	12	13
D7S820	7	8
D16S539	11	12
CSF1PO	11	13
Penta D	8	8
D2S441	11	14
vWA	16	16
D8S1179	13	16
TPOX	8	11
Penta E	14	14
TH01	6	6
D12S391	21	21
D2S1338	23	23
FGA	23	23

CCTCC

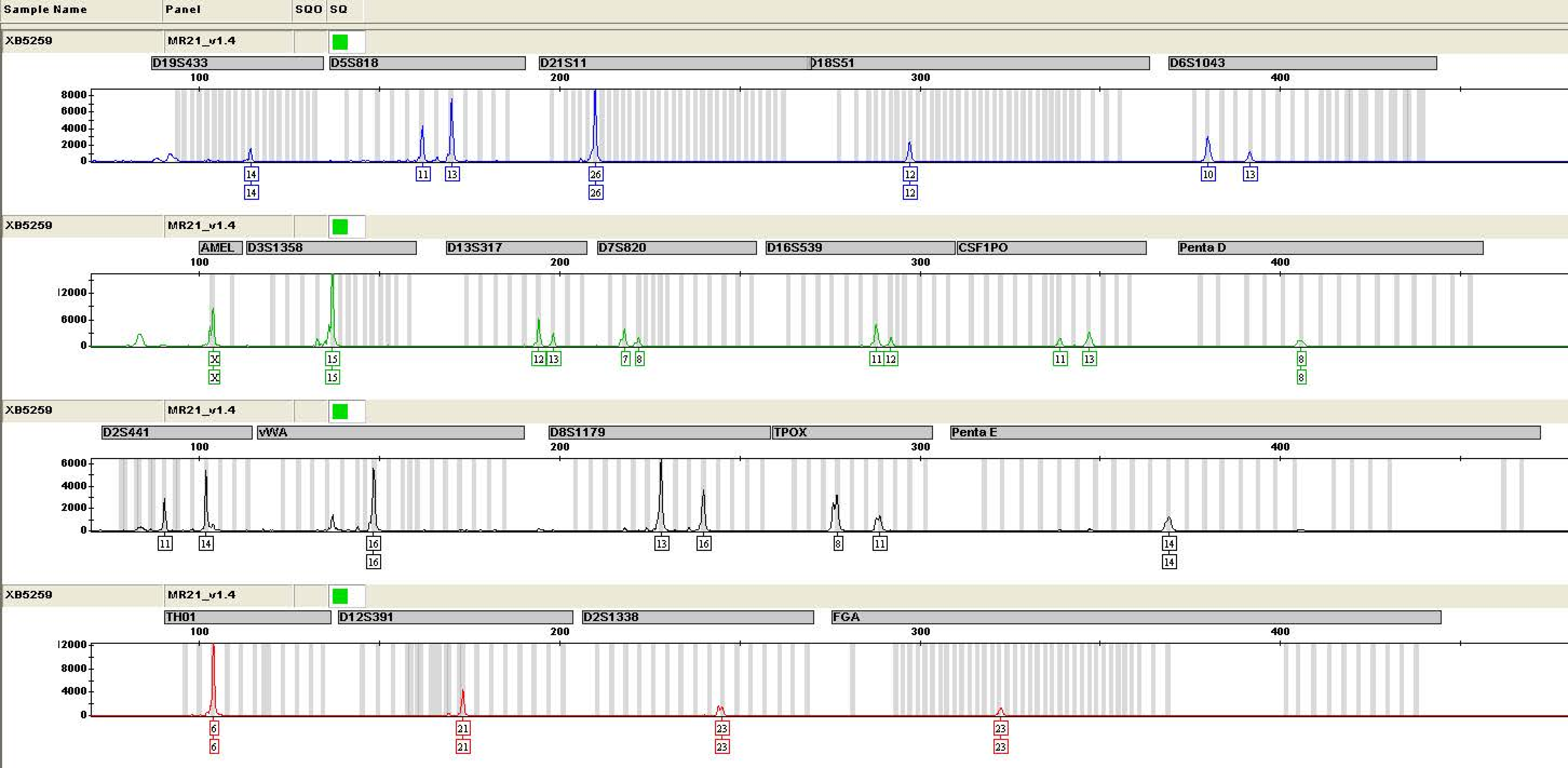
Sample Name
Panel
SQO
SQ
XB5259
MR21_v1.4
D19S433
D5S818
D21S11
D18S51
D6S1043
100
200
300
400
8000
6000
4000
2000
0
14
11
13
26
12
10
XB5259
MR21_v1.4
AMEL
D3S1358
D13S317
D7S820
D16S539
CSF1PO
Penta D
12000
6000
X
15
12
13
7
8
11
XB5259
MR21_v1.4
D2S441
vWA
D8S1179
TPOX
Penta E
16
XB5259
MR21_v1.4
TH01
D12S391
D2S1338
FGA
6
21
23
