# Supplementary material for: TOB1 suppresses proliferation in K‐Ras wild‐type pancreatic cancer
Source: Cancer Med. 2019 Dec 31;9(4):1503–14. doi: 10.1002/cam4.2756 (PMC7013073; doi:10.1002/cam4.2756)
Supplement: Supplementary file 11 [file CAM4-9-1503-s011.pdf]

中国典型培养物保藏中心
CHINA CENTER FOR TYPE CULTURE COLLECTION (CCTCC)
Wuhan University, Wuhan 430072, China
Phone: 86-027-68752093 **Fax:** 86-027-68754833 **Email:** shenchao@whu.edu.cn

3-3-2017

Entrusted by the Second Affiliated Hospital of Xi'an Jiaotong University, CCTCC has conducted identification experiments on the SW1990 cell line, and come to the following conclusions:

1. There was no third allele found in SW1990 cell line, it indicating that there was no cross-contaminant of human source cell line.
2. Compared the STR data of SW1990 cell line in the databases of ATCC and DSMZ, all the locations of SW1990 were exactly matched with the locations of SW 1990 (Pancreatic Carcinoma Human) cells found in ATCC and DSMZ cell banks, so it is SW 1990 (Pancreatic Carcinoma Human) cell line (Table 1).

Manager:

China Center for Type Culture Collection

Table 1. The alleles of 21 locations in SW1990 cell line

SW1990 cell line（Fig. No.XB5262）		
Marker	Allele 1	Allele 2
D19S433	13	15
D5S818	12	13
D21S11	31	31
D18S51	13	13
D6S1043	11	19
AMEL	X	X
D3S1358	16	16
D13S317	8	12
D7S820	9	10
D16S539	13	13
CSF1PO	10	12
Penta D	11	11
D2S441	11	15
vWA	17	17
D8S1179	11	14
TPOX	8	9
Penta E	5	7
TH01	9.3	9.3
D12S391	17	17
D2S1338	23	23
FGA	26	26

CCTCC

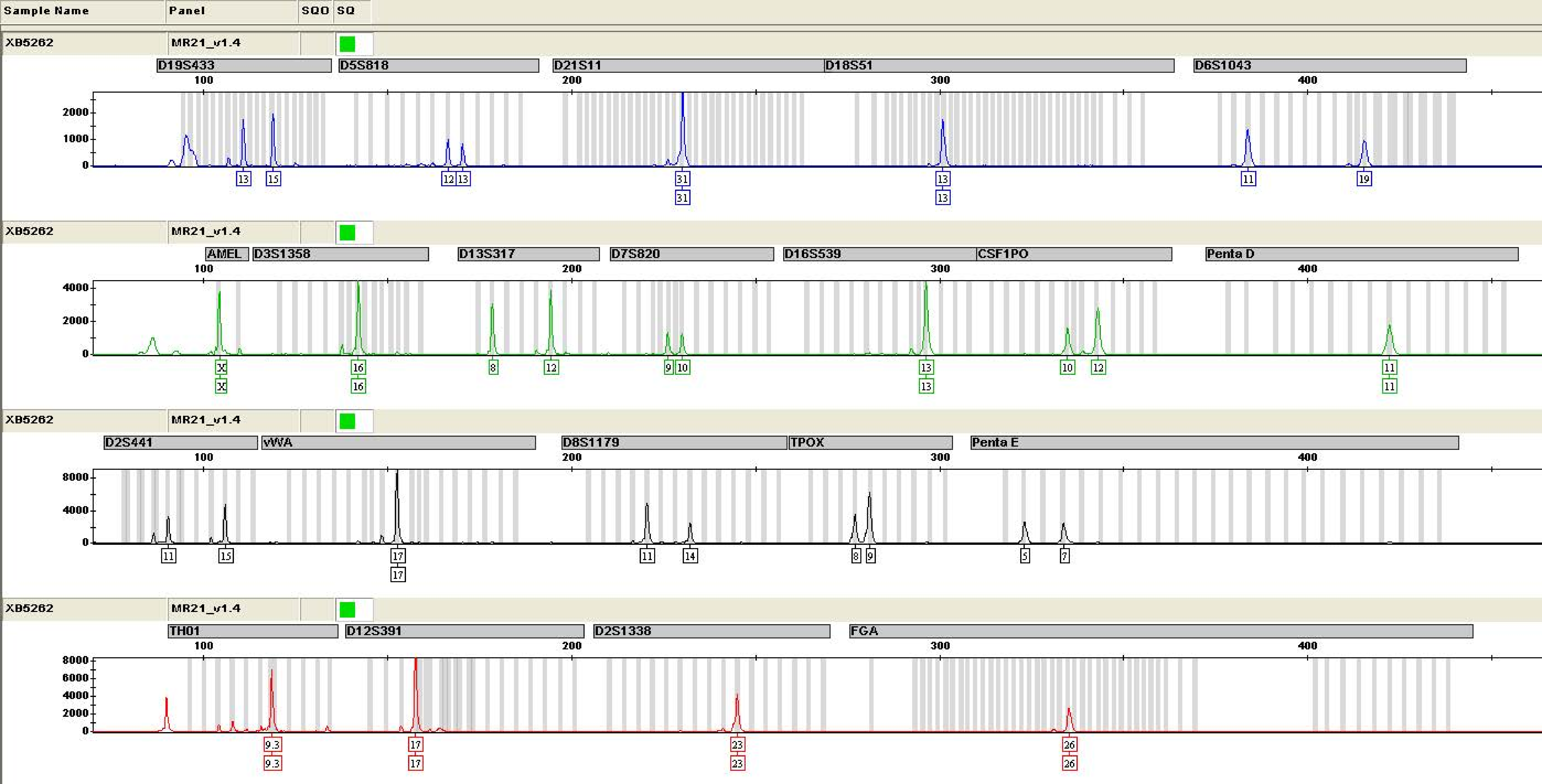

Sample Name
Panel
SQO
SQ
XB5262
MR21_v1.4
D19S433
D5S818
D21S11
D18S51
D6S1043
13
15
12
13
31
31
13
13
11
19
XB5262
MR21_v1.4
AMEL
D3S1358
D13S317
D7S820
D16S539
CSF1PO
Penta D
X
X
16
16
8
12
9
10
13
13
10
12
11
11
XB5262
MR21_v1.4
D2S441
vWA
D8S1179
TPOX
Penta E
11
15
17
17
11
14
8
9
5
7
XB5262
MR21_v1.4
TH01
D12S391
D2S1338
FGA
9.3
9.3
17
17
23
23
26
26
