# Supplementary material for: TOB1 suppresses proliferation in K‐Ras wild‐type pancreatic cancer
Source: Cancer Med. 2019 Dec 31;9(4):1503–14. doi: 10.1002/cam4.2756 (PMC7013073; doi:10.1002/cam4.2756)
Supplement: Supplementary file 12 [file CAM4-9-1503-s012.pdf]

中国典型培养物保藏中心

CHINA CENTER FOR TYPE CULTURE COLLECTION (CCTCC)

Wuhan University, Wuhan 430072, China

Phone: 86-027-68752093 **Fax:** 86-027-68754833 **Email:** shenchao@whu.edu.cn

10-26-2017

Entrusted by The Second Affiliated Hospital of Xi'an Jiaotong University, CCTCC has conducted identification experiments on the Bxpc-3 cell line, and come to the following conclusions:

1. There was no third allele found in Bxpc-3 cell line, it indicating that there was no cross-contaminant of human source cell line.
2. Compared the STR data of Bxpc-3 cell line in the databases of ATCC and DSMZ, its profile does not exactly match with any of the current data (Table 1).
3. The STR data of Bxpc-3 cell line and BxPC-3 cell line matches the highest rate of 94% in DSMZ database.

Manager:

China Center for Type Culture Collection

Table 1. The alleles of 21 locations in Bxpc-3 cell line

Bxpc-3 cell line（Fig. No.XB6447）		
Marker	Allele 1	Allele 2
D19S433	13	16.2
D5S818	11	11
D21S11	29	29
D18S51	12	12
D6S1043	12	12
AMEL	X	X
D3S1358	14	16
D13S317	11	11
D7S820	10	13
D16S539	11	11
CSF1PO	13	13
Penta D	14	14
D2S441	12	14
vWA	14	18
D8S1179	13	13
TPOX	8	8
Penta E	12	14
TH01	9	9
D12S391	19.3	20
D2S1338	17	19
FGA	20	21

CCTCC

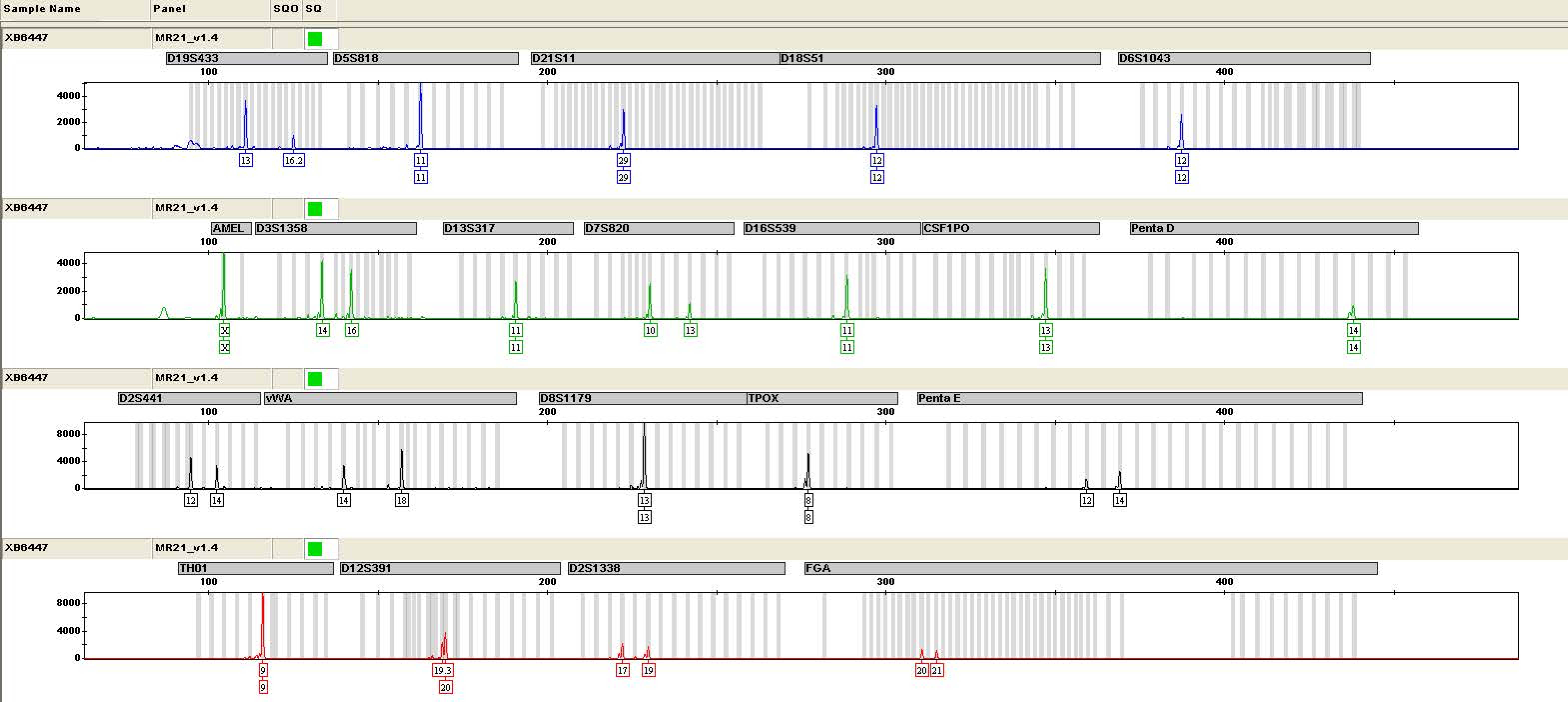
Sample Name
Panel
SQO
SQ
XB6447
MR21_v1.4
D19S433
D5S818
D21S11
D18S51
D6S1043
100
200
300
400
4000
2000
0
13
16.2
11
11
29
29
12
12
12
12
XB6447
MR21_v1.4
AMEL
D3S1358
D13S317
D7S820
D16S539
CSF1PO
Penta D
X
X
14
16
11
11
10
13
11
11
13
13
14
14
XB6447
MR21_v1.4
D2S441
vWA
D8S1179
TPOX
Penta E
8000
4000
0
12
14
14
18
13
13
8
8
12
14
XB6447
MR21_v1.4
TH01
D12S391
D2S1338
FGA
9
9
19.3
20
17
19
20
21
