# Supplementary material for: TOB1 suppresses proliferation in K‐Ras wild‐type pancreatic cancer
Source: Cancer Med. 2019 Dec 31;9(4):1503–14. doi: 10.1002/cam4.2756 (PMC7013073; doi:10.1002/cam4.2756)
Supplement: Supplementary file 13 [file CAM4-9-1503-s013.pdf]

中国典型培养物保藏中心

CHINA CENTER FOR TYPE CULTURE COLLECTION (CCTCC)

Wuhan University, Wuhan 430072, China

Phone: 86-027-68752093 **Fax:** 86-027-68754833 **Email:** shenchao@whu.edu.cn

3-2-2017

Entrusted by the Second Affiliated Hospital of Xi'an Jiaotong University, CCTCC has conducted identification experiments on the CFPAC-1 cell line, and come to the following conclusions:

1. There was no third allele found in CFPAC-1 cell line, it indicating that there was no cross-contaminant of human source cell line.
2. Compared the STR data of CFPAC-1 cell line in the databases of ATCC and DSMZ, all the locations of CFPAC-1 were exactly matched with the locations of HCT-15 (Colorectal Carcinoma Human) cells found in ATCC and DSMZ cell banks, so it is HCT-15 (Colorectal Carcinoma Human) cell line (Table 1).

Manager：

China Center for Type Culture Collection

Table 1. The alleles of 21 locations in CFPAC-1 cell line

CFPAC-1 cell line（Fig. No.XB5261）		
Marker	Allele 1	Allele 2
D19S433	14	16
D5S818	13	13
D21S11	29	32.2
D18S51	17	17
D6S1043	11	13
AMEL	X	Y
D3S1358	17	17
D13S317	8	11
D7S820	10	12
D16S539	12	13
CSF1PO	12	12
Penta D	9	14
D2S441	13	15
vWA	18	19
D8S1179	15	15
TPOX	8	11
Penta E	7	14
TH01	7	9.3
D12S391	19	22
D2S1338	17	25
FGA	22	22

CCTCC

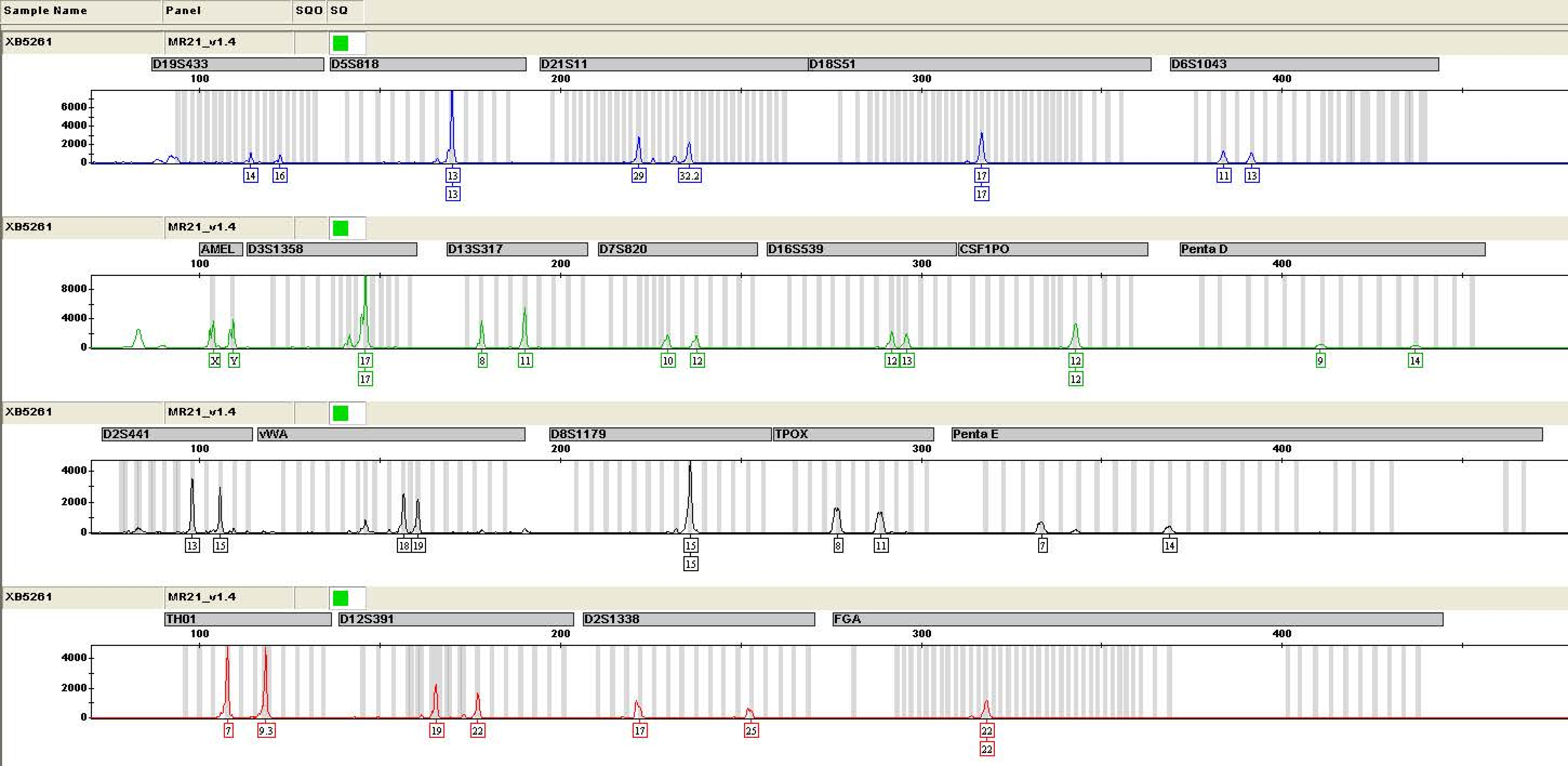
Sample Name
Panel
SQO
SQ
XB5261
MR21_v1.4
D19S433
D5S818
D21S11
D18S51
D6S1043
14 16 13 13 29 32.2 17 17 11 13
XB5261
MR21_v1.4
AMEL
D3S1358
D13S317
D7S820
D16S539
CSF1PO
Penta D
X Y 17 17 8 11 10 12 12 13 12 12 9 14
XB5261
MR21_v1.4
D2S441
vWA
D8S1179
TPOX
Penta E
13 15 18 19 15 15 8 11 7 14
XB5261
MR21_v1.4
TH01
D12S391
D2S1338
FGA
7 9.3 19 22 17 25 22 22
