# Supplementary material for: TOB1 suppresses proliferation in K‐Ras wild‐type pancreatic cancer
Source: Cancer Med. 2019 Dec 31;9(4):1503–14. doi: 10.1002/cam4.2756 (PMC7013073; doi:10.1002/cam4.2756)
Supplement: Supplementary file 14 [file CAM4-9-1503-s014.pdf]

中国典型培养物保藏中心
CHINA CENTER FOR TYPE CULTURE COLLECTION (CCTCC)
Wuhan University, Wuhan 430072, China
Phone: 86-027-68752093 **Fax:** 86-027-68754833 **Email:** shenchao@whu.edu.cn

3-2-2017

Entrusted by the Second Affiliated Hospital of Xi'an Jiaotong University, CCTCC has conducted identification experiments on the PANC-1 cell line, and come to the following conclusions:

1. There was no third allele found in PANC-1 cell line, it indicating that there was no cross-contaminant of human source cell line.
2. Compared the STR data of PANC-1 cell line in the databases of ATCC and DSMZ, all the locations of PANC-1 were exactly matched with the locations of HeLa (Cervical Adenocarcinoma Human) cells found in ATCC and DSMZ cell banks, so it is HeLa (Cervical Adenocarcinoma Human) cell line (Table 1).

Manager:

China Center for Type Culture Collection

中国典型培养物保藏中心
业务专用章

Table 1. The alleles of 21 locations in PANC-1 cell line

PANC-1 cell line（Fig. No.XB5260）		
Marker	Allele 1	Allele 2
D19S433	13	14
D5S818	11	12
D21S11	27	28
D18S51	16	16
D6S1043	18	18
AMEL	X	X
D3S1358	15	18
D13S317	12	13.3
D7S820	8	12
D16S539	9	10
CSF1PO	9	10
Penta D	8	15
D2S441	10	11
vWA	16	18
D8S1179	13	13
TPOX	8	12
Penta E	7	17
TH01	7	7
D12S391	20	25
D2S1338	17	17
FGA	18	21

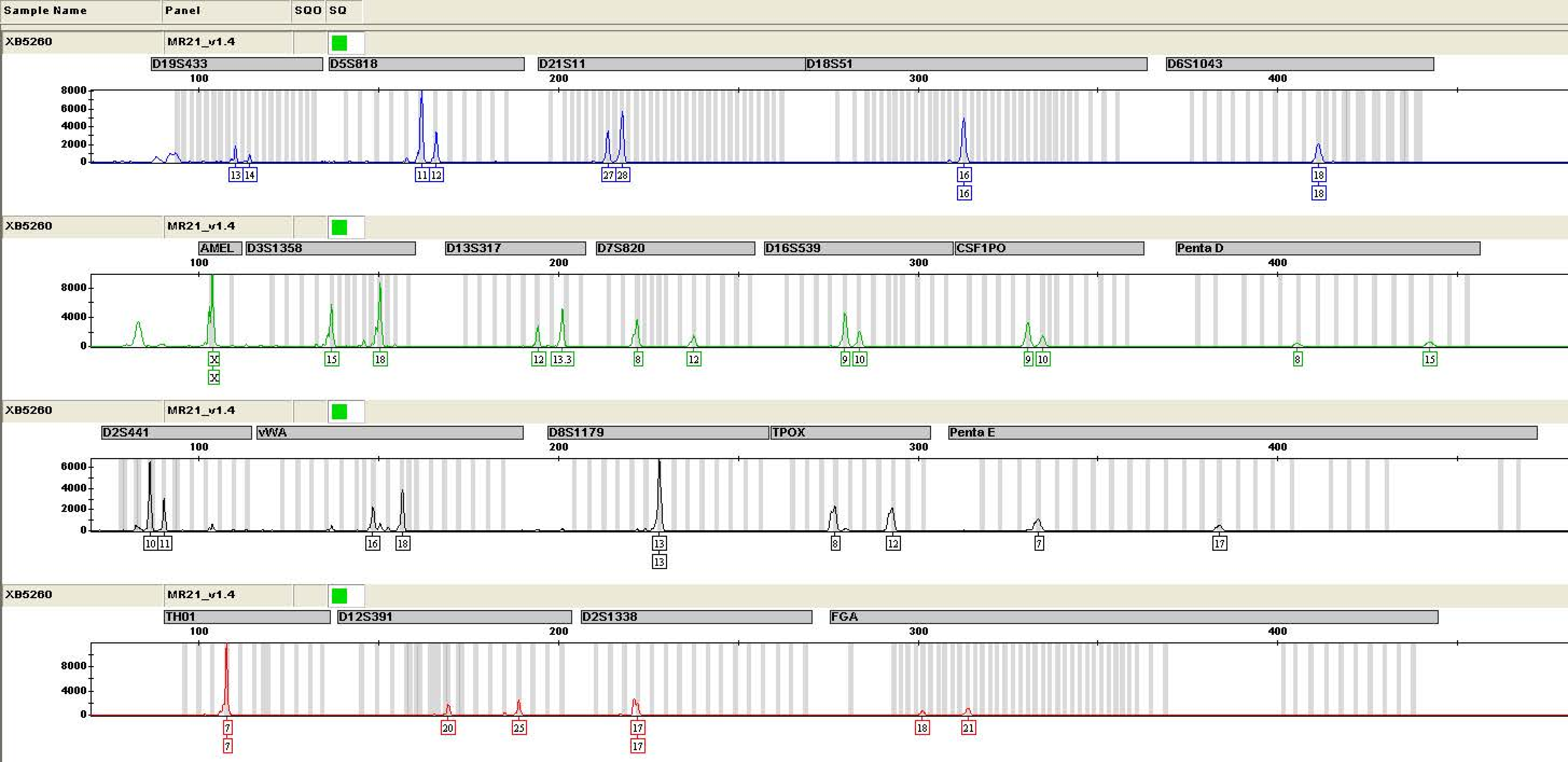

Sample Name
Panel
SQO
SQ
XB5260
MR21_v1.4
D19S433
D5S818
D21S11
D18S51
D6S1043
100
200
300
400
8000
6000
4000
2000
0
13
14
11
12
27
28
16
16
18
18
XB5260
MR21_v1.4
AMEL
D3S1358
D13S317
D7S820
D16S539
CSF1PO
Penta D
8000
4000
X
X
15
18
12
13.3
8
12
9
10
9
10
8
15
XB5260
MR21_v1.4
D2S441
vWA
D8S1179
TPOX
Penta E
6000
4000
2000
10
11
16
18
13
13
8
12
7
17
XB5260
MR21_v1.4
TH01
D12S391
D2S1338
FGA
8000
4000
7
7
20
25
17
17
18
21
