# Supplementary material for: TOB1 suppresses proliferation in K‐Ras wild‐type pancreatic cancer
Source: Cancer Med. 2019 Dec 31;9(4):1503–14. doi: 10.1002/cam4.2756 (PMC7013073; doi:10.1002/cam4.2756)
Supplement: Supplementary file 15 [file CAM4-9-1503-s015.docx]

NM_001243877-promoter TOB1

TGTTATTTGCTCAGAGAGAGGAATACCTCTTGATTGTGATACACTTGGAGCCTAATATCAAATTCTTTTTTCCTCCATAAGTCTAATTCCCTGCACAATTAGCAGTTAGCCCTCCACTCCCCTGCACAATTCCAAAGTAAATGTTAGATAATAATAAGCCATATGCAGAACTTGGATAAAGAACATTTTATACCTCGTGACTGGCAATGTTCAGAGATGTGAATCAGTTTTCCCTTTTATTTGCAATCCATATATATGTATTTTTTTTCTTTTTTGAAACGAAGTTTTGCTCCTGTTGCCTAGGCTGGAGTGCAATGGCGCGATCCCAGCTCACCGCAACCTCCGCCTCCCGGGTTCAAGTGATTCTCCTGCCTCAGCCTCCCGAGTAGCTGGGATTACAGGCATGCGCCACCATGCCAGGCTAATTTTGTATTTTTAGTAGAGACAGGGTTTCTCCATGTTGGTCAAGCTGATCTCGAGCTCCCGACCTCAGATGATCCACCCACCTTGGCCTCCCAAAGTACTGGGATTACAGGCGAGAGCCACCGCGCCCTGACTTGCAATCTGTATTTGAAGAACAGCTGCAGTATATATGTTAGGAACATTGAGTTTTAAGGGCGGAATATTTTATTTATTTATTTTTTTGAGAGAGAGTCTCGCTCTGTCGGCCAGCAGGAGTGCAGTGGCACGATCGCGGCGCACTGCAACCTCCATCTCCCAGGCTCAAGCAATTCTCCTGCCTCAGCCTCCCAAGTAGCTGGGATTACAGGCGTGTGCCACTACACCCGGCTAATTTTTGTATTTTCAGTAGAGACGGGGTTTCACCGTGTTGGCCAGGCTGGTCTCAAATTCCTGAGCTCAGGTAATCTGCCTGCCTCAGCCTCCCAAAGTGCTGGGATTACAGGCGTAAGCCACTGCTCCCAGCCAAGTGTGGAATTTTTTTTTAATTGAAGGAGACATTAGATATGATCTAATTCAACCCTTTTTATAGGTGAGAAAACTAAAAGTTTATTGAAACTGGTGTGAAACTCAGACCTCCCAGTACCCACCCTTCATTGCTTTTTCCAGTATGCCATGGGGCCTCCGTGTGTGTTGTTTGTGTGGTGTGTGTGTGTGTGTTTATATTTGGTCAGACATCTTCTTACACTCCTAGGCCAAGCAGCAATAACTCATAAATTTTTGACATCTACATTTCTGACTTAAAAATGCTTTTAATGCTACTGATATTTAATCAGTACCTATCAGATGACTGCTCTCATATTTCAAATCTGGAATTACAGATTGAAGACATTCAGAAAAGAGAACTTGTCCCTTCTCTTTAGAGAATTATTGAAAAGTACAGATAAGTTTGACAAAATTACAATTCATCTATCTTTAGACATTTTAGTCTCAGCCATGCCTAATATGCCCCCCCAATATAAAATTACTAATCAAGACACATGGTCCCGCAGTTCTAGATGTTTGGTACAGTACCATTATTTGCCTAATGGTAAAAGAAGTTGTGCTTCTAGAACACGTGAAAAATGAAGTGTTTCTTTTGGTAAGGCATTCCAAGGATATCCTGTCTAGTTTCTTGAAATTTCTGTGCTTAATTAGAAAACTAAAAATAACTCACTTTTATTACAGATGGACTTGTCATTACAGAAAACCAAAAACAAACAAATAAAAAAAGAAAACTGAACAAATCATTTATATTTGACGCTTTGATGACATTTCAATGCAGAGGAAGGGACATTTTAATTTAAATGCTTTCACAGTTTTGCCATACTTTCACATTTCAATTCCACTCCTGTTTGCAACTAAAAAACCCAAACACTCAAAAAAAAAAAAAAATCACATTAATCAGAATTTAAGGAGTCACTTAGTAAAACTATGTTTTAGGAATCTGTACAATAAACATGAAATCAGTCACCTGACCACTCCCCACCCTCAGCCCATTCCACAAACCCATCTTCCCACCCCTTCTCCTACGCATTGAGCCTTCAGAAATACCCTTTCTCAGTGCGGTTCCTTCTAAAAAGGAGAGGCGGGAGAGTCAGGGTGGAGAAGG
