# Supplementary material for: TOB1 suppresses proliferation in K‐Ras wild‐type pancreatic cancer
Source: Cancer Med. 2019 Dec 31;9(4):1503–14. doi: 10.1002/cam4.2756 (PMC7013073; doi:10.1002/cam4.2756)
Supplement: Supplementary file 16 [file CAM4-9-1503-s016.doc]

**Supplementary Figure 1. Efficiency validation of lentivirus transfection preliminary experiment**

(A) RPMI-1640 medium group. (B) RPMI-1640 medium containing 5 μg/ml Polybrene group. (C) Enhanced infection solution group. (D) Enhanced infection solution containing 5 μg/ml Polybrene group. Red boxes represent the conditions used for formal experiment. All images were taken at ×200 magnification.

**Supplementary Figure 2. Correlation between *TOB1* mRNA expression and overall survival in TCGA-PAAD tissues**

**Supplementary Figure 3. Location of TOB1 protein in pancreatic cancer cells**

DAPI, stained nucleus. TOB1, antibody labeled with Alexa Fluor 594. Merge is a fusion location. B, Bxpc-3; B-NC, BxPC-3-LV-NC; B-T, BxPC-3-LV-TOB1; P, Patu8988t; P-NC, Patu8988t-LV-NC; P-T, Patu8988t-LV-TOB1. All images were taken at ×200 magnification.
